# Conjugates of Iron-Transporting *N*-Hydroxylactams with Ciprofloxacin

**DOI:** 10.3390/molecules27123910

**Published:** 2022-06-18

**Authors:** Olga Bakulina, Anton Bannykh, Ekaterina Levashova, Mikhail Krasavin

**Affiliations:** 1 Saint Petersburg State University, 26 Universitetskii Prospect, 198504 Peterhof, Russia; o.bakulina@spbu.ru (O.B.); bannykh.anton.v@gmail.com (A.B.); levashovaekaterinayu@gmail.com (E.L.); 2 Immanuel Kant Baltic Federal University, 236041 Kaliningrad, Russia

**Keywords:** Castagnoli-Cushman reaction, cyclic hydroxamic acids, siderophores, bacterial iron transport, Trojan horse antibiotics

## Abstract

Screening of a library of novel *N*-hydroxylactams amenable by the Castagnoli-Cushman reaction identified four lead compounds that facilitated ^55^Fe transport into *P. aeruginosa* cells (one of these synthetic siderophores was found to be as efficient at promoting iron uptake as the natural siderophores pyoverdine, pyochelin or enterobactin). Conjugates of the four lead siderophores with ciprofloxacin were tested for antibacterial activity against *P. aeruginosa* POA1 (wild type) and the *∆pvdF∆pchA* mutant strain. The antibacterial activity was found to be pronounced against the *∆pvdF∆pchA* mutant strain grown in CAA medium but not for the POA1 strain. This may be indicative of these compounds being ‘Trojan horse’ antibiotics. Further scrutiny of the mechanism of the antibacterial action of the newly developed conjugates is warranted.

## 1. Introduction

**Figure 1:**
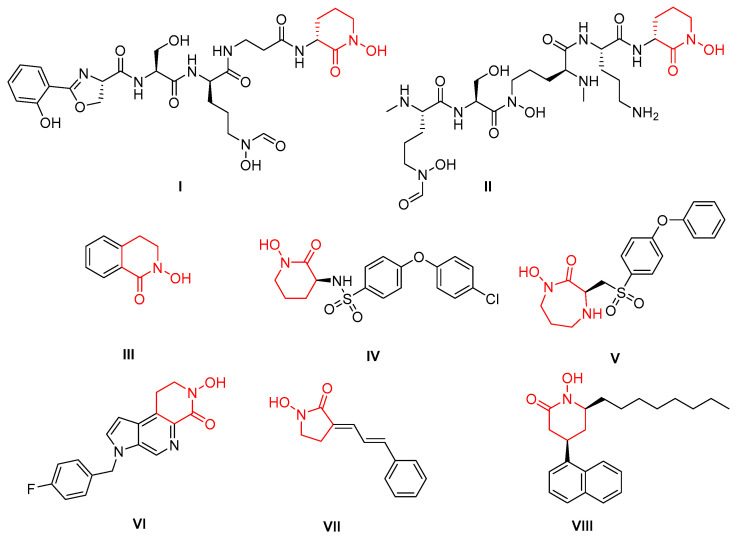
Examples of *N*-hydroxylactams whose biological activity is exerted via metal chelation.

Cyclic hydroxamic acid (*N*-hydroxylactam) moieties are frequently encountered in natural products and synthetic inhibitors of therapeutically relevant enzyme biotargets. The biological activity of *N*-hydroxylactams often has to do with their ability to chelate metal ions. Chelation of iron is the principal mechanism of action of microbial siderophores (metabolites excreted by iron-starved procaryotic microorganisms into extracellular space to scavenge iron needed for such processes as growth and differentiation [1,2]), examples of which include oxachelin (**I**) [3], scabichelin (**II**) [4] as well as structurally simple *N*-hydroxy-3,4-dihydroisoquinolin-1-one (**III**) which was isolated from *Streptomyces griseus* [5]. Anchoring to the prosthetic metal ion is central to the activity of small-molecule *N*-hydroxylactam inhibitors of such important zinc enzyme targets as matrix metalloprotease (e.g., **IV** [6] and **V** [7]), HIV integrase (e.g., **VI** [8]), histone deacetylases (e.g., **VII** [9]) as well as of (non-heme) iron-containing enzyme lipoxygenase (e.g., **VIII** or BMD 188 [10]) (Figure 1).

Some biological effects of *N*-hydroxylactams manifest themselves independently of metal chelation. Indeed, bacterial siderophores have been shown to possess a signaling function, antibacterial activity and to regulate oxidative stress [11]. At the same time, iron-loaded bacterial siderophores and their synthetic analogs were dubbed ‘Trojan horse’ carriers for antibiotics and fluorescent labels [12,13]. Conjugation of antibiotics or a fluorescent drugs otherwise eliminated by efflux-mediated resistance mechanisms to a siderophore molecule is a promising approach to circumventing drug resistance as well as to in vivo imaging and eradication of bacterial colonies [14]. This approach received clinical proof-of-concept in the 2019 approval of cefiderocol (Fetroja) for the treatment of complicated urinary tract infections [15].

**Scheme 1:**
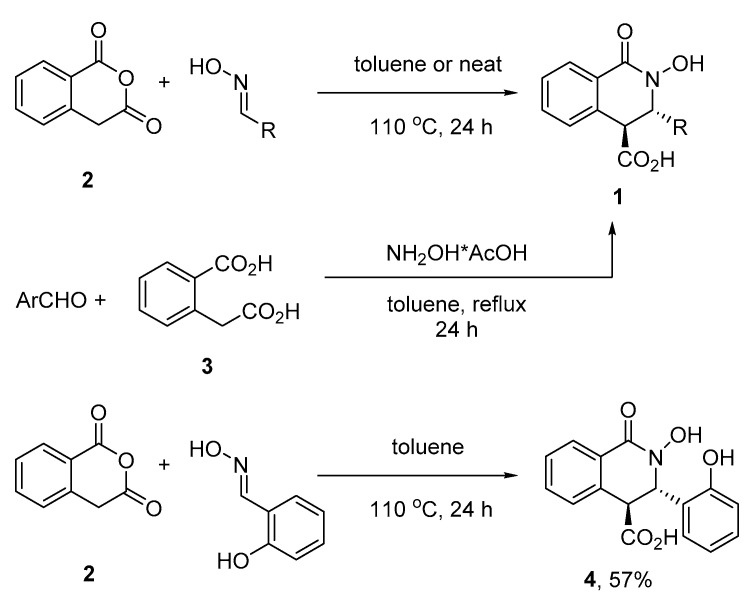
Preparation of racemic *trans*-2-hydroxy-1-oxo-3-aryl-1,2,3,4-tetrahydroisoquinoline-4-carboxylic acids **1** and **4** (relative stereochemistry shown).

Recently, we developed a facile access to a novel type of *N*-hydroxylactams (cyclic hydroxamic acids)—*trans*-2-hydroxy-1-oxo-3-aryl-1,2,3,4-tetrahydroisoquinoline-4-carboxylic acids **1**—via a two-component condensation of oximes with homophthalic anhydride (**2**) [16,17] or via a three-component condensation of aromatic aldehydes, hydroxylamine acetate and homophthalic acid (**3**) with azeotropic removal of water [18]. In addition to twenty-six hydroxamic acids **1a–z** prepared in the course of this investigation (for structures—see Supplementary Materials), we have recently prepared 2-hydroxy-3-(2-hydroxyphenyl)-1-oxo-1,2,3,4-tetrahydroisoquinoline-4-carboxylic acid (**4**) as a compound capable of providing an additional phenolic hydroxy group for metal chelation (Scheme 1).

Having amassed the library of novel *N*-hydroxylactams **1a–z** and **4**, we became interested in identifying, via screening, non-cytotoxic lead compounds capable of transporting iron into Gram-negative *Pseudomonas aeruginosa* bacterial cells. We reasoned that, considering that these compounds contain a conveniently positioned carboxylic acid functionality, the newly identified lead iron-complexing—and iron-transporting-compounds could be conjugated to a known antibiotic molecule (e.g., ciprofloxacin as was recently described [19,20]). Herein, we report our results obtained in the course of realization of this strategy.

## 2. Results and Discussion

In order to establish if *P. aeruginosa* cells are able to use any of the synthesized compounds **1a–z** or **4** to access extracellular iron, we used a growth assay in iron-restricted conditions, i. e. using iron-deficient casamino acid (CAA) medium and iron concentration around 20 nM, according to the previous work [21]. The *P. aeruginosa ∆pvdF∆pchA* mutant unable to produce the siderophores pyoverdine [22] and pyochelin [23] was used [24] and was grown on a CAA medium in the absence or presence of 10 mM and 100 mM concentration of the compounds screened. This bacterial strain is able to grow in an iron-deficient environment likely due to citrate acting as siderophore [25] or owing to the functioning of the iron reduction systems which import ferrous ions through the *feoABC* system [26]. The assay is based on the premise that in the presence of an excess of a metal chelator, all iron traces present in the growth medium become scavenged, making this nutrient no longer available for low-affinity iron import systems. However, if the growth of the *P. aeruginosa ∆pvdF∆pchA* mutant is still observed in the presence of the tested compounds, it would be indicative of the bacteria being able to use these iron chelators as siderophores.

A large portion of compounds **1a–z** and **4** tested displayed nearly total growth inhibition at 10 μM, indicating that such compounds cannot be used by *P. aeruginosa* as siderophores to access iron. For 12 compounds tested (**1a**, **1c–e**, **1i–k**, **1t–v**, **1x**, **1z**, **4**), the results were more promising, with a total inhibition at 100 μM and no or a partial inhibition at 10 μM (see Figure S1, Supplementary Materials). These compounds were then screened for the ability to transport ^55^Fe into *P. aeruginosa* cells.

**Figure 2:**
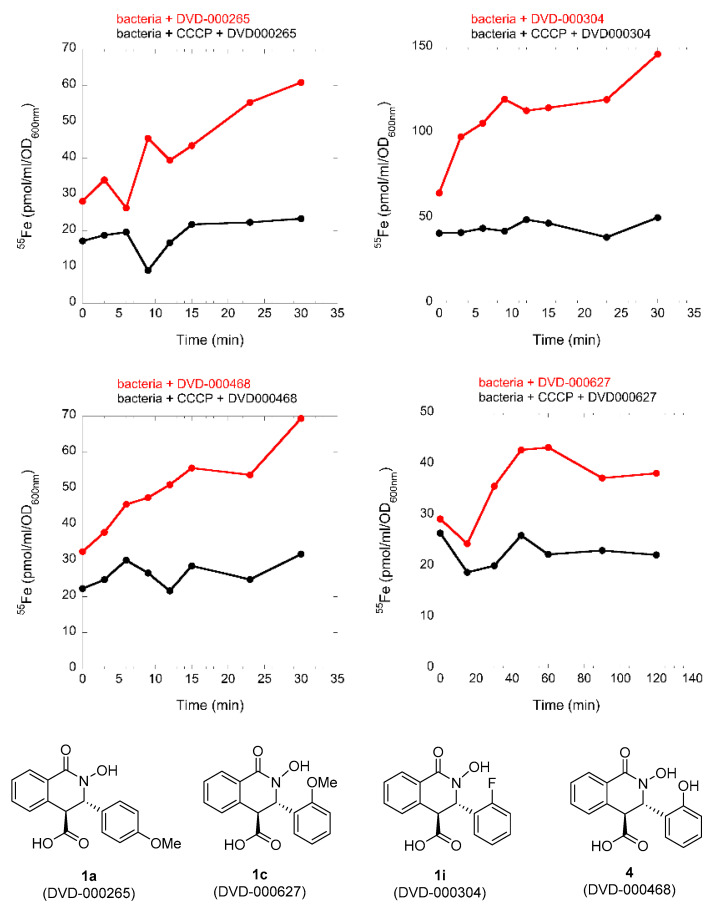
Transport of ^55^Fe via compounds **1a** (DVD-000265), **1c** (DVD-00627), **1i** (DVD-000304 and **4** (DVD-000468) in *P. aeruginosa* cells; structures of the four lead siderophores; *P. aeruginosa* pyoverdine and pyochelin deficient strain (*∆pvdF∆pchA*) was grown in CAA medium overnight as described in Materials and Methods. Afterwards, cells were washed with 50 mM Tris-HCl pH 8.0 and re-suspended in the same buffer to and OD_600 nm_ (optical density measured at 600 nm) of 1. The iron transport assay was started by the addition of 500 nM ^55^Fe-loaded compounds **1a**, **1c**, **1i** and **4** and, at different time points, aliquots were taken, bacteria were pelleted and the radioactivity retained in the cells was monitored. The *∆pvdF∆pchA* strain was also treated with 200 mM of CCCP protonophore and incubated in the presence of ^55^Fe-loaded compounds (CCCP inhibits any proton motive dependent uptake).

The compounds were loaded with ^55^Fe and incubated with the *P. aeruginosa ∆pvdF∆pchA* mutant. The latter was chosen so as to avoid any uptake of ^55^Fe via the endogenous siderophores (pyoverdine and pyochelin). Reassuringly, for compounds **1a** (DVD-000265), **1c** (DVD-00627) and **4** (DVD-000468), an uptake of around 40–70 pmol of ^55^Fe/mL/OD_600 nm_ after 30 min was observed and only in the absence of the protonophore carbonyl cyanide *m*-chlorophenyl hydrazone (CCCP) [27]. Because CCCP had been shown to inhibit any iron uptake by TonB-dependent transporters (bacterial outer membrane proteins that bind and transport siderophores [28]) in bacteria [29,30], this finding indicates that the uptake of iron by these compounds was not due to passive diffusion via porins [31], but rather due to a proton motive force-dependent transport relying on TonB. A special case was identified for compound **1i** (DVD-000304), as an uptake of about 150 pmol ^55^Fe/mL/OD_600 nm_ was observed after 30 min incubation and was also abolished in the presence of CCCP indicating it being a TonB-dependent uptake. Such a high ^55^Fe uptake by compound **1i** was judged to be as efficient as the one observed for natural siderophores pyoverdine, pyochelin or enterobactin in *P. aeruginosa* [30]. Altogether, these data show an iron uptake into *P. aeruginosa* cells in the presence of compounds **1a** (DVD-000265), **1c** (DVD-00627), **1i** (DVD-000304 and **4** (DVD-000468), which suggests that these compounds can be used as siderophores by this pathogen (Figure 2).

**Scheme 2:**
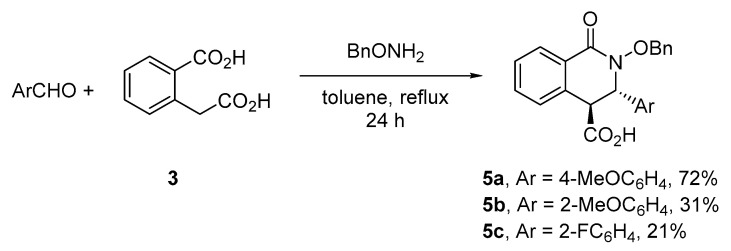
Synthesis of *N*-benzyloxylactams **5a–c**.

Having identified the four lead synthetic siderophores (**1a**, **1c**, **1i** and **4**), all of which contain a carboxylic acid functionality suitable for conjugation, we proceeded to synthesize the ‘Trojan horse’-type conjugates of these siderophores with antibiotic ciprofloxacin. To this end, we synthesized three *N*-benzyloxylactams **5a–c** as depicted in Scheme 2.

**Scheme 3:**
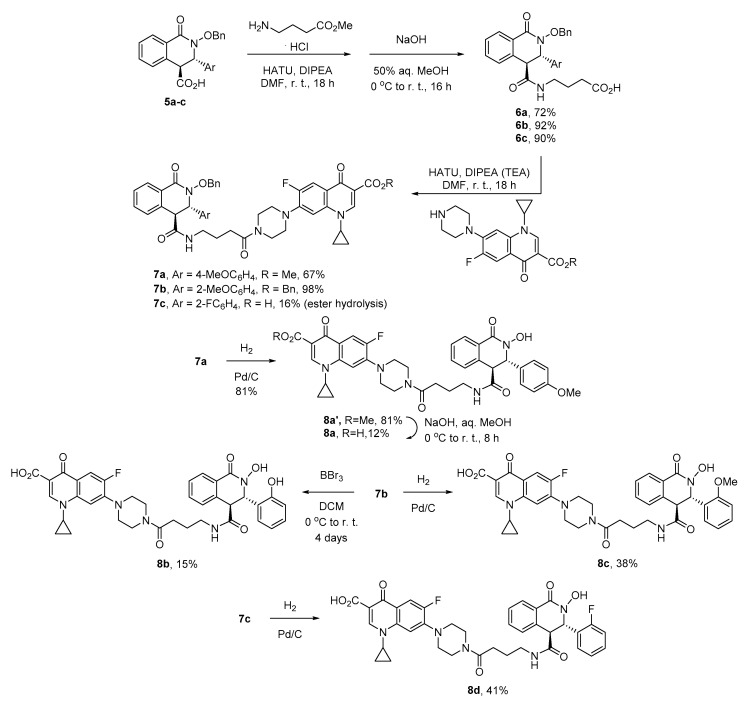
Synthesis of ciprofloxacin-siderophore conjugates **8a–d**.

Compounds **5a–c** were amidated using HATU and methyl 4-aminobutanoate [32]. Following basic hydrolysis, carboxylic acid building blocks **6a–c** were obtained in excellent yields over two steps. HATU-mediated coupling with ciprofloxacin methyl or benzyl ester gave compounds **7a–c** (in case of **7c**, the benzyl ester group underwent hydrolysis during the HPLC purification). Removal of the benzyl group from the *N*-benzyloxy lactam moiety and ester hydrolysis of intermediate **8a’** gave compound **8a**. Treatment of compound **7b** with BBr_3_ led to the simultaneous deprotection of the *N*-hydroxylactam, the ester and the phenolic hydroxy group to give compound **8b**. Hydrogenation of **7b** and **7c** gave siderophore-ciprofloxacin conjugates **8c** and **8d**, respectively (Scheme 3).

Siderophore-ciprofloxacin conjugates **8a–d** were tested for antibacterial activity against *P. aeruginosa* PAO1 (wild type) strain and the *∆pvdF∆pchA* mutant. Since the latter are not able to produce pyoverdine and pyochelin, they may be able to more efficiently use the synthetic siderophore-ciprofloxacin conjugates to get access to iron compared to the PAO1 strain. The minimum inhibitory concentrations (MIC) were first determined for both strains grown in Müller-Hinton (MH) medium [33]. For both strains, no antibacterial activity was detected, which is not surprising for bacteria grown in MH medium since the outer membrane transporters involved in iron acquisition are only expressed under iron-restricted growth conditions [30]. Consequently, we also determined the MIC values in iron-restricted conditions (CAA medium). Again, no antibiotic activity was observed against the PAO1 strain. However, for the *∆pvdF∆pchA* mutant, MICs of 1 mM were determined for all four siderophore-ciprofloxacin conjugates (Table 1).

The observed antibacterial effect from siderophore-ciprofloxacin conjugates **8a–d** solely on the *P. aeruginosa ∆pvdF∆pchA* mutant strain grown in (iron-restricted) medium may be indicative of these conjugates indeed acting as ‘Trojan horse’ antibiotics. The iron-starved bacteria unable to produce and excrete their own siderophores (pyoverdine and pyochelin) are likely to utilize the siderophoric constructs **8a–d** to shuttle the iron inside the cells. Once inside the bacteria, these constructs may already exert their antibiotic activity due to the presence of a ciprofloxacin payload. Unfortunately, we were unable to monitor the ^55^Fe uptake with these compounds because of the solubility problems encountered.

## 3. Materials and Methods

### 3.1. Bacterial Strains and Growth Assays in Iron-Restricted Conditions

The *P. aeruginosa* PAO1 and *∆pvdF∆pchA* strains were used in this study. PAO1 is a wild type strain and ∆pvdF∆pchA is a strain unable to produce the siderophores pyoverdine and pyochelin [22]. Bacteria were first grown in LB medium overnight at 30 °C, and then washed and re-suspended in 20 mL iron-deficient CAA (casamino acid) medium containing 5 g∙L^−1^ low-iron CAA (Difco), 1.46 g∙L^−1^ K_2_HPO_4_∙3H_2_O and 0.25 g∙L^−1^ MgSO_4_∙7H_2_O and grown overnight at 30 °C. Bacteria were then washed, re-suspended in CAA medium at an optical density of 0.02 at 600 nm and distributed into the wells of a 96-well plate (Greiner, U-bottomed microplate) in the absence or in the presence of 10 µM or 100 µM of the tested compounds (5 mM stock solutions in DMSO were used). The plate was incubated at 30 °C, with shaking, in a TECAN microplate reader (Infinite M200, Tecan) (Tecan, Männedorf, Switzerland) and bacterial growth was monitored at OD_600 nm_. The presented data are the mean of three replicates for each measurement.

### 3.2. Iron Uptake

Compound-^55^Fe complexes were prepared at ^55^Fe concentrations of 50 µM, with a compound:iron (mol:mol) ratio of 200:1 as described previously [22]. *P. aeruginosa* strains were successively grown an overnight in LB broth, followed by an overnight in CAA medium and finally overnight in CAA medium. All of these successive cultures were carried out at 30 °C. The bacteria were subsequently used for ^55^Fe uptake kinetics as described previously [22], with a concentration of ^55^Fe-compound complexes of 500 nM and with cells pretreated or not with 200 µM CCCP (a proton motive force inhibitor) [27].

### 3.3. MIC Determination

Overnight cultures were grown at 37 °C in Lysogeny broth (LB) and diluted to obtain an opacity equivalent to 0.5 on the McFarland scale. Screening vials were filled with solutions of the test compounds in 0.5% DMSO as prepared above with three replications for each treatment. Ciprofloxacin (0.5–256 µg/mL) and 0.5% DMSO served as positive and negative controls, respectively. The entire vial was incubated at 35 ± 2 °C for 18 h. After incubation, the antibacterial activity of the test compounds was determined by measuring the absorption of the solution with a spectrophotometer on 500 nm.

The MICs were measured using the twofold serial broth dilution method. The test organisms were grown in suitable broth for 18 h at 37 °C. Twofold serial dilutions of solutions of the test compounds were prepared at 256, 128, 64, 32, 16, 8, 4, 2, 1 and 0.5 µg ml^−1^. The tubes were then inoculated with the test microbe; each 5 mL received 0.1 mL of the above inoculums and were incubated at 37 °C. The vials were subsequently observed for the presence or absence of microbial growth.

### 3.4. Compounds Synthesis

NMR spectra were acquired with a 400 MHz Bruker Avance III spectrometer (400.13 MHz for ^1^H, 376.49 MHz for ^19^F and 100.61 MHz for ^13^C) or 500 MHz Bruker Avance III (500.03 MHz for ^1^H and 125.73 MHz for ^13^C) in CDCl_3_ or DMSO-*d_6_* and were referenced to residual solvent proton signals (δ_H_ = 7.26 and 2.50, respectively) and solvent carbon signals (δ_C_ = 77.16 and 39.52, respectively). Multiplicities are abbreviated as follows: s = singlet, d = doublet, t = triplet, q = quartet, m = multiplet, br = broad, dd = doublet of doublets, dt = doublet of triplets, ddd = doublet/doublets of doublets; coupling constants, J, are reported in Hz. Mass spectra were acquired with an HRMS-ESI-qTOF spectrometer Nexera LCMS9030 (Shimadzu Europa GmbH, Duisburg, Germany) or MaXis II Bruker Daltonic GmbH (electrospray ionization mode, positive ions detection) (Bremen, Germany). Flash column chromatography on silica (Merck, 230–400 mesh) was performed with a Biotage Isolera Prime instrument. TLC was performed on aluminum-backed pre-coated plates (0.25 mm) with silica gel 60 F_254_ with a suitable solvent system and was visualized using UV fluorescence. Preparative HPLC was carried out in a compact preparative system ECOM ECS28P00 equipped with a spectrophotometric detector or a Shimadzu LC-20AP. Column: YMC-Pack SIL-06, 5 µm, 250 × 20 mm or Agilent Zorbax prepHT XDB-C18, 5 µm, 21.2 × 150 mm. Chlorobenzene was distilled from P_2_O_5_ and stored over molecular sieves 4 Å (>24 h). Compound **5a** was prepared as described previously [18].

#### 3.4.1. (3*RS*,4*RS*)-2-Hydroxy-3-(2-hydroxyphenyl)-1-oxo-1,2,3,4-tetrahydroisoquinoline-4-carboxylic acid (**4**)

A mixture of homophthalic anhydride (162 mg, 1 mmol) and 2-hydroxybenzaldehyde oxime (137 mg, 1 mmol) was refluxed in toluene (2 mL) for 16 h in a screw-cap vial under stirring. After cooling to room temperature, filtration of the solid and washing it with ether afforded 170 mg, 57% of pure title compound as white solid. ^1^H NMR (400 MHz, DMSO-*d*_6_) δ 12.95 (s, 1H), 10.15 (s, 1H), 9.90 (s, 1H), 8.00–7.91 (m, 1H), 7.43–7.34 (m, 2H), 7.30–7.21 (m, 1H), 7.08–6.97 (m, 1H), 6.84 (d, *J* = 7.9 Hz, 1H), 6.65–6.51 (m, 2H), 5.71 (s, 1H), 4.15 (s, 1H). ^13^C NMR (101 MHz, DMSO-*d*_6_) δ 172.1, 160.7, 154.3, 133.1, 131.6, 130.0, 128.5, 128.5, 127.8, 126.5, 125.8, 124.0, 118.6, 115.3, 60.7, 49.1. HRMS m/z [M+Na]^+^ calculated for C_16_H_13_NO_5_Na^+^ 322.0686, found 322.0682.

#### 3.4.2. (3*RS*,4*RS*)-2-(Benzyloxy)-3-(2-methoxyphenyl)-1-oxo-1,2,3,4-tetrahydroisoquinoline-4-carboxylic acid (**5b**)

A mixture of homophthalic anhydride (320 mg, 2 mmol) and 2-methoxybenzaldehyde *O*-benzyl oxime (482 mg, 1 mmol) was refluxed in toluene (2 mL) for 72 h in a screw-cap vial under stirring. Cooling to room temperature, filtration of the solid and washing it with ether afforded 250 mg, 31% of pure title compound as white solid. ^1^H NMR (400 MHz, CDCl_3_ + DMSO-*d_6_*) δ 13.17 (s, 1H), 7.99 (dd, *J* = 7.4, 1.8 Hz, 1H), 7.51–7.40 (m, 4H), 7.40–7.28 (m, 4H), 7.25–7.17 (m, 1H), 7.04 (dd, *J* = 8.3, 1.0 Hz, 1H), 6.78–6.71 (m, 1H), 6.66 (dd, *J* = 7.8, 1.7 Hz, 1H), 5.95 (d, *J* = 1.7 Hz, 1H), 5.02 (s, 2H), 4.24 (d, *J* = 1.7 Hz, 1H), 3.89 (s, 3H). ^13^C NMR (101 MHz, DMSO-*d_6_*) δ 171.8, 161.4, 156.2, 135.0, 133.3, 132.3, 130.2, 129.2, 129.1, 128.5, 128.3, 128.0, 128.0, 126.8, 125.7, 125.2, 120.1, 111.4, 75.9, 59.1, 55.7, 49.5. HRMS m/z [M+Na]^+^ calculated for C_24_H_21_NO_5_^+^ 426.1312, found 426.1305.

#### 3.4.3. (3*RS*,4*RS*)-2-(Benzyloxy)-3-(2-fluorophenyl)-1-oxo-1,2,3,4-tetrahydroisoquinoline-4-carboxylic acid (**5c**)

A mixture of homophthalic acid (3.6 g, 20 mmol), *O*-benzylhydroxylamine (2.5 g, 20 mmol) and 2-fluorobenzaldehyde (2.5 g, 20 mmol) was suspended in toluene (20 mL) and refluxed under stirring for 48 h with the azeotropic removal of water (Dean-Stark head). Cooling to room temperature, filtration of the solid and its crystallization from ACN afforded 1.65 g, 21% of pure title compound as white solid. ^1^H NMR (400 MHz, CDCl_3_) δ 13.28 (s, 1H), 8.06–7.99 (m, 1H), 7.56–7.19 (m, 10H), 7.03 (t, *J* = 7.4 Hz, 1H), 6.84 (t, *J* = 7.4 Hz, 1H), 6.00 (s, 1H), 5.05 (d, *J* = 10.0 Hz, 1H), 5.00 (d, *J* = 10.0 Hz, 1H), 4.35 (d, *J* = 1.6 Hz, 1H). ^13^C NMR (101 MHz, CDCl_3_) δ 171.8, 161.9, 160.1 (d, *J* = 245.3 Hz), 135.4, 133.6, 133.0, 130.6, 129.8, 129.7, 129.1, 128.8, 128.7, 128.3, 127.5 (d, *J* = 3.6 Hz), 127.4, 125.5 (d, *J* = 13.1 Hz), 125.0 (d, *J* = 3.1 Hz), 116.4 (d, *J* = 21.0 Hz), 76.5, 58.8 (d, *J* = 3.0 Hz), 50.6. HRMS m/z [M+Na]^+^ calculated for C_23_H_18_NFO_4_Na^+^ 414.1112, found 414.1109.

#### 3.4.4. 4-((3*RS*,4*RS*)-2-(Benzyloxy)-3-(4-methoxyphenyl)-1-oxo-1,2,3,4-tetrahydroisoquinoline-4-carboxamido)butanoic acid (**6a**)

To a solution of rac-(3*R*,4*R*)-2-(benzyloxy)-3-(4-methoxyphenyl)-1-oxo-1,2,3,4-tetrahydroisoquinoline-4-carboxylic acid (**5a**, 500 mg, 1.2 mmol) in DMF (2 mL) DIPEA (351 mg, 2.7 mmol) and HATU (518 mg, 1.4 mmol) were added at room temperature. After stirring for 5 min, methyl 4-aminobutanoate hydrochloride (201 mg, 1.4 mmol) was added and the mixture was left overnight. The reaction mixture was diluted with DCM and washed with water. The organic phase was dried over sodium sulfate, filtered and concentrated to give a crude product which was purified by flash column chromatography on silica (mobile phase CHCl_3_-MeOH 25:1) to give 486 mg of methyl 4-((3*RS*,4*RS*)-2-(benzyloxy)-3-(4-methoxyphenyl)-1-oxo-1,2,3,4-tetrahydroisoquinoline-4-carboxamido)butanoate, which was taken into the next step without full characterization. The obtained material was dissolved in aqueous MeOH (5 mL, 50%), cooled to 0 °C and treated with KOH (108 mg, 1.94 mmol). After stirring at room temperature for 16 h, the reaction mixture was acidified to pH 1 with HCl conc. and partitioned between EtOAc (20 mL) and water (10 mL). The aqueous layer was extracted with EtOAc (10 mL) and combined organics were dried over sodium sulfate, filtered and concentrated to give pure title compound in 72% yield (422 mg) as white foam. ^1^H NMR (400 MHz, CDCl_3_) δ 8.28–8.21 (m, 1H), 7.53–7.47 (m, 2H), 7.46–7.38 (m, 2H), 7.36–7.28 (m, 3H), 7.18–7.11 (m, 1H), 7.06 (d, *J* = 8.8 Hz, 2H), 6.73 (d, *J* = 8.8 Hz, 2H), 5.83 (d, *J* = 2.1 Hz, 1H), 5.47 (t, *J* = 5.9 Hz, 1H), 5.05 (d, *J* = 9.9 Hz, 1H), 5.01 (d, *J* = 10.0 Hz, 1H), 3.95 (d, *J* = 2.2 Hz, 1H), 3.70 (s, 3H), 3.32–3.20 (m, 2H), 2.25 (t, *J* = 6.9 Hz, 2H), 1.71 (p, *J* = 6.8 Hz, 2H). ^13^C NMR (101 MHz, DMSO-*d*_6_) δ 174.1, 169.6, 161.0, 158.8, 135.2, 134.4, 132.5, 130.8, 129.0, 128.8, 128.4, 128.2, 128.1, 127.8, 127.6, 127.1, 113.8, 79.2, 75.6, 63.7, 55.0, 52.5, 38.3, 38.2, 30.9, 24.4. HRMS m/z [M+Na]^+^ calculated for C_28_H_28_N_2_O_6_Na^+^ 511.1840, found 511.1840.

#### 3.4.5. 4-((3*RS*,4*RS*)-2-(Benzyloxy)-3-(2-methoxyphenyl)-1-oxo-1,2,3,4-tetrahydroisoquinoline-4-carboxamido)butanoic acid (**6b**)

This compound was obtained following the same two-step procedure as for compound **6a** starting from (3*RS*,4*RS*)-2-(benzyloxy)-3-(2-methoxyphenyl)-1-oxo-1,2,3,4-tetrahydroisoquinoline-4-carboxylic acid (**5b**, 845 mg, 2.1 mmol) in 92% yield (941 mg). ^1^H NMR (400 MHz, CDCl_3_) δ 8.28–8.22 (m, 1H), 7.51–7.42 (m, 4H), 7.34–7.27 (m, 3H), 7.20–7.12 (m, 1H), 7.12–7.06 (m, 1H), 6.89–6.80 (m, 2H), 6.71 (td, *J* = 7.6, 1.0 Hz, 1H), 6.21 (d, *J* = 1.9 Hz, 1H), 5.45 (t, *J* = 5.9 Hz, 1H), 5.10 (d, *J* = 9.9 Hz, 1H), 5.04 (d, *J* = 10.0 Hz, 1H), 4.14 (d, *J* = 2.0 Hz, 1H), 3.91 (s, 2H), 3.35–3.18 (m, 2H), 2.26 (t, *J* = 7.0 Hz, 2H), 1.78–1.66 (m, 2H). ^13^C NMR (101 MHz, CDCl_3_) δ 176.8, 170.5, 162.9, 156.5, 135.1, 133.2, 133.0, 129.6, 129.6, 129.2, 129.1, 129.0, 129.0, 128.6, 128.4, 126.8, 125.2, 120.7, 110.7, 60.4, 55.6, 51.8, 39.6, 38.8, 31.3, 24.4. HRMS m/z [M+Na]^+^ calculated for C_28_H_28_N_2_O_6_Na^+^ 511.1840, found 511.1841.

#### 3.4.6. 4-((3*RS*,4*RS*)-2-(Benzyloxy)-3-(2-fluorophenyl)-1-oxo-1,2,3,4-tetrahydroisoquinoline-4-carboxamido)butanoic acid (**6c**)

This compound was obtained following the same two-step procedure as for compound **6a** starting from (3*RS*,4*RS*)-2-(benzyloxy)-3-(2-fluorophenyl)-1-oxo-1,2,3,4-tetrahydroisoquinoline-4-carboxylic acid (**5c**, 782 mg, 2.0 mmol) in 90% yield (856 mg). ^1^H NMR (400 MHz, CDCl_3_) δ 8.26 (dd, *J* = 5.8, 3.5 Hz, 1H), 7.54–7.43 (m, 4H), 7.36–7.28 (m, 3H), 7.21–7.12 (m, 2H), 7.07–6.98 (m, 1H), 6.94–6.86 (m, 2H), 6.24 (d, *J* = 2.0 Hz, 1H), 5.44 (t, *J* = 5.9 Hz, 1H), 5.11 (d, *J* = 10.0 Hz, 1H), 5.05 (d, *J* = 10.0 Hz, 1H), 4.07 (d, *J* = 2.0 Hz, 1H), 3.28 (q, *J* = 6.8 Hz, 2H), 2.27 (t, *J* = 6.9 Hz, 2H), 1.79–1.67 (m, 2H). ^13^C NMR (101 MHz, CDCl_3_) δ 176.4, 169.7, 162.7, 160.2 (d, *J* = 246.3 Hz), 134.9, 133.4, 132.4, 129.8 (d, *J* = 8.3 Hz), 129.6, 129.4, 129.2, 129.1, 129.0, 128.8, 128.5, 127.7 (d, *J* = 3.4 Hz), 124.9, 124.8, 124.5 (d, *J* = 3.5 Hz), 115.9 (d, *J* = 21.2 Hz), 59.6 (d, *J* = 2.5 Hz), 52.7 (d, *J* = 2.2 Hz), 39.8, 31.2, 24.3. HRMS m/z [M+Na]^+^ calculated for C_27_H_25_FN_2_O_5_Na^+^ 499.1640, found 499.1644.

#### 3.4.7. Methyl 7-(4-(4-((3*RS*,4*RS*)-2-(benzyloxy)-3-(4-methoxyphenyl)-1-oxo-1,2,3,4-tetrahydroisoquinoline-4-carboxamido)butanoyl)piperazin-1-yl)-1-cyclopropyl-6-fluoro-4-oxo-1,4-dihydroquinoline-3-carboxylate (**7a**)

To a solution of compound **6a** (100 mg, 0.2 mmol), DMF (1 mL) DIPEA (60 mg, 0.47 mmol) and HATU (84 mg, 0.22 mmol) were added at room temperature. After stirring for 5 min, methyl methyl 1-cyclopropyl-6-fluoro-4-oxo-7-(piperazin-1-yl)-1,4-dihydroquinoline-3-carboxylate hydrochloride (76 mg, 0.2 mmol) was added and the mixture was left overnight. The reaction mixture was diluted with EtOAc and washed with water. The organic phase was dried over sodium sulfate, filtered and concentrated to give the crude product which was purified by flash column chromatography on silica (mobile phase hex-acetone 5–100%, then acetone-MeOH) to give 113 mg, 67% of pure title compound. ^1^H NMR (400 MHz, DMSO-*d*_6_) δ 8.45 (s, 1H), 8.31 (t, *J* = 5.7 Hz, 1H), 7.98 (dd, *J* = 7.7, 1.5 Hz, 1H), 7.78 (d, *J* = 13.3 Hz, 1H), 7.54–7.48 (m, 1H), 7.47–7.38 (m, 2H), 7.34–7.28 (m, 6H), 7.17–7.10 (m, 2H), 6.87–6.79 (m, 2H), 5.44 (d, *J* = 3.1 Hz, 1H), 5.00 (d, *J* = 9.8 Hz, 1H), 4.90 (d, *J* = 9.9 Hz, 1H), 4.20 (d, *J* = 3.2 Hz, 1H), 3.74 (s, 3H), 3.71–3.68 (m, 1H), 3.67 (s, 3H), 3.65–3.59 (m, 2H), 3.57–3.47 (m, 2H), 3.26–3.07 (m, 6H), 2.28 (t, *J* = 7.3 Hz, 2H), 1.70–1.57 (m, 2H), 1.29–1.19 (m, 2H), 1.12–1.02 (m, 2H). ^13^C NMR (101 MHz, DMSO-*d*_6_) δ 170.9 (d, *J* = 133.2 Hz), 169.7, 164.9, 160.7, 158.8, 152.6 (d, *J* = 246.9 Hz), 148.3, 143.6 (d, *J* = 10.3 Hz), 138.0, 135.1, 134.5, 132.4, 130.9, 129.0, 128.8, 128.3 (d, *J* = 20.3 Hz), 128.2, 127.8, 127.6, 127.0, 126.9, 122.1, 122.0, 113.8, 111.6 (d, *J* = 22.3 Hz), 109.0, 106.5, 75.6, 63.5, 55.0, 52.3, 51.2, 49.8, 49.3, 44.6, 40.7, 38.4, 34.7, 29.6, 29.3, 24.6, 7.5. HRMS m/z [M+Na]^+^ calculated for C_46_H_46_FN_5_O_8_Na^+^ 838.3223, found 838.3255.

#### 3.4.8. Benzyl 7-(4-(4-((3*RS*,4*RS*)-2-(benzyloxy)-3-(2-methoxyphenyl)-1-oxo-1,2,3,4-tetrahydroisoquinoline-4-carboxamido)butanoyl)piperazin-1-yl)-1-cyclopropyl-6-fluoro-4-oxo-1,4-dihydroquinoline-3-carboxylate (**7b**)

To a solution of compound **6b** (630 mg, 1.15 mmol) in DCM (20 mL) NEt_3_ (0.5 mL) and HATU (480 mg, 1.26 mmol) were added at room temperature. After stirring for 5 min, benzyl 1-cyclopropyl-6-fluoro-4-oxo-7-(piperazin-1-yl)-1,4-dihydroquinoline-3-carboxylate (550 mg, 1.31 mmol) was added and the mixture was left stirring overnight. The reaction mixture was diluted with water (30 mL) + conc. HCl (1 mL) and the resulting suspension was filtered. The solid material was washed with water (30 × 3 mL) and ether (20 × 2 mL) and dried in air to give pure title compound. Yield 1.00 g, 98%. ^1^H NMR (400 MHz, CDCl_3_) δ 8.66 (s, 1H), 8.26–8.16 (m, 1H), 8.05 (d, *J* = 13.0 Hz, 1H), 7.47 (d, *J* = 4.1 Hz, 4H), 7.44–7.30 (m, 6H), 7.29–7.21 (m, 3H), 7.20–7.10 (m, 2H), 6.90–6.78 (m, 2H), 6.71 (t, *J* = 7.5 Hz, 1H), 6.20 (d, *J* = 1.8 Hz, 1H), 6.05 (t, *J* = 5.5 Hz, 1H), 5.38 (s, 2H), 5.07 (d, *J* = 10.1 Hz, 1H), 5.02 (d, *J* = 10.1 Hz, 1H), 4.13 (d, *J* = 1.8 Hz, 1H), 3.91 (s, 3H), 3.77 (s, 1H), 3.72 (s, 1H), 3.65–3.53 (m, 3H), 3.40–3.21 (m, 6H), 2.40–2.19 (m, 2H), 1.89–1.69 (m, 2H), 1.38 (d, *J* = 6.9 Hz, 2H), 1.18–1.08 (m, 2H). 177.2, 171.3, 170.7, 167.4, 162.6, 156.4, 153.7 (d, *J* = 251.5 Hz), 147.7, 145.5 (d, *J* = 10.4 Hz), 140.9, 139.2, 135.0, 133.3, 133.1, 129.4, 129.3, 129.0, 128.7, 128.7, 128.5, 128.4, 127.8, 127.1, 126.7, 125.1, 125.1, 120.7, 120.3 (d, *J* = 7.9 Hz), 112.7, 112.5, 110.7, 108.1, 105.5 (d, *J* = 2.5 Hz), 65.4, 60.3, 55.6, 51.8, 51.8, 50.0 (d, *J* = 4.7 Hz), 49.6, 45.4, 41.6, 40.2, 35.6, 30.5, 24.1, 8.4, 8.4. HRMS m/z [M+Na]^+^ calculated for C_52_H_50_FN_5_O_8_Na^+^ 914.3536, found 914.3533.

#### 3.4.9. 7-(4-(4-((3*SR*,4*SR*)-2-(Benzyloxy)-3-(2-fluorophenyl)-1-oxo-1,2,3,4-tetrahydroisoquinoline-4-carboxamido)butanoyl)piperazin-1-yl)-1-cyclopropyl-6-fluoro-4-oxo-1,4-dihydroquinoline-3-carboxylic acid (**7c**)

To a solution of compound **6c** (240 mg, 0.5 mmol) in DCM (20 mL) DIPEA (195 mg, 1.5 mmol) and HATU (210 mg, 0.55 mmol) were added at room temperature. After stirring for 5 min, benzyl 1-cyclopropyl-6-fluoro-4-oxo-7-(piperazin-1-yl)-1,4-dihydroquinoline-3-carboxylate hydrochloride (230 mg, 0.5 mmol) was added and the mixture was left stirring overnight. The reaction mixture was washed with water and HCl 1N. The organic phase was separated, dried and concentrated to give crude product, which was purified by flash column chromatography (DCM-MeOH) go give 333 mg of product with ~80% purity. This sample was further purified by preparative RP-HPLC (ACN-water + 0.1%TFA; gradient 20–90% of ACN in 35 min; 40 °C, 12 mL/min) to give 65 mg, 16% of pure title compound. The hydrolysis of the benzyl ester moiety probably occurred during HPLC separation. ^1^H NMR (400 MHz, DMSO-*d*_6_) δ 8.62 (s, 1H), 8.09 (t, *J* = 5.7 Hz, 1H), 8.03 (d, *J* = 7.6 Hz, 1H), 7.87 (d, *J* = 13.0 Hz, 1H), 7.60–7.44 (m, 3H), 7.42–7.19 (m, 8H), 7.08 (d, *J* = 7.4 Hz, 1H), 7.03 (d, *J* = 7.6 Hz, 1H), 5.77 (d, *J* = 3.4 Hz, 1H), 5.06 (d, *J* = 9.8 Hz, 1H), 4.90 (d, *J* = 9.8 Hz, 1H), 4.21 (d, *J* = 3.4 Hz, 1H), 3.85–3.59 (m, 4H), 3.53 (s, 2H), 3.28 (s, 3H), 3.22–3.07 (m, 2H), 2.25 (t, *J* = 7.2 Hz, 2H), 1.65 (q, *J* = 7.3 Hz, 2H), 1.31 (d, *J* = 6.9 Hz, 2H), 1.23–1.09 (m, 2H). ^13^C NMR (101 MHz, DMSO-*d*_6_) δ 176.8 (d, *J* = 2.3 Hz), 170.7, 169.8, 166.3, 161.4, 160.2 (d, *J* = 244.8 Hz), 153.4 (d, *J* = 249.1 Hz), 148.4, 145.3 (d, *J* = 10.2 Hz), 139.5, 135.4, 134.5, 133.3, 130.6 (d, *J* = 7.9 Hz), 129.5, 128.9, 128.7, 128.6, 128.1, 127.8, 126.0 (d, *J* = 12.6 Hz), 125.0, 119.2 (d, *J* = 7.7 Hz), 116.3 (d, *J* = 21.1 Hz), 111.4 (d, *J* = 23.0 Hz), 107.2, 106.9, 76.2, 58.8, 51.4, 50.0, 49.6, 44.9, 41.1, 38.9, 36.3, 29.7, 25.0, 8.0.. HRMS m/z [M+Na]^+^ calculated for C_44_H_41_F_2_N_5_O_7_Na^+^ 812.2866, found 812.2872.

#### 3.4.10. 1-Cyclopropyl-6-fluoro-7-(4-(4-((3*RS*,4*RS*)-2-hydroxy-3-(4-methoxyphenyl)-1-oxo-1,2,3,4-tetrahydroisoquinoline-4-carboxamido)butanoyl)piperazin-1-yl)-4-oxo-1,4-dihydroquinoline-3-carboxylic acid (**8a**)

In a round bottom flask equipped with a rubber septum and magnetic stir bar, compound **7a** (180 mg, 0.22 mmol) was dissolved in THF/MeOH mixture (10/30 mL) followed by the addition of Pd/C (10% wt) as catalyst (180 mg, 0.75 eq). Hydrogen gas (1 atm) was slowly bubbled through the reaction mixture via needle for 4 h and then the hydrogen inlet was removed and the reaction mixture was left stirring under atmosphere of hydrogen overnight. The reaction progress was monitored via HPLC. After completion of the reaction the reaction mixture was filtered through a pad of Celite, which was washed with MeOH 20 mL and hot DMF 50 mL. The filtrate was concentrated and the residue was treated with DCM-Et_2_O mixture (1:3) under ultrasound. After cooling to −20 °C the resulting solid was filtered to give 131 mg, 81% of methyl 1-cyclopropyl-6-fluoro-7-(4-(4-((3*RS*,4*RS*)-2-hydroxy-3-(4-methoxyphenyl)-1-oxo-1,2,3,4-tetrahydroisoquinoline-4-carboxamido)butanoyl)piperazin-1-yl)-4-oxo-1,4-dihydroquinoline-3-carboxylate: ^1^H NMR (400 MHz, DMSO-*d*_6_) δ 9.98 (s, 1H), 8.45 (s, 1H), 8.08 (t, *J* = 5.7 Hz, 1H), 7.93 (d, *J* = 1.6 Hz, 1H), 7.78 (d, *J* = 13.3 Hz, 1H), 7.48–7.36 (m, 3H), 7.21 (dd, *J* = 7.5, 1.4 Hz, 1H), 7.11–7.05 (m, 2H), 6.86–6.79 (m, 2H), 5.18 (d, *J* = 2.9 Hz, 1H), 4.04 (d, *J* = 3.0 Hz, 1H), 3.74 (s, 3H), 3.71–3.68 (m, 1H), 3.68 (s, 3H), 3.66–3.61 (m, 2H), 3.60–3.53 (m, 2H), 3.20 (d, *J* = 6.6 Hz, 4H), 3.12 (q, *J* = 7.6, 7.2 Hz, 2H), 2.29 (t, *J* = 7.3 Hz, 2H), 1.74–1.58 (m, 2H), 1.28–1.21 (m, 2H), 1.13–1.04 (m, 2H). ^13^C NMR (126 MHz, DMSO-*d*_6_) δ 171.6, 170.3, 169.7, 164.9, 160.3, 158.6, 152.6 (d, *J* = 246.8 Hz), 148.3, 143.6 (d, *J* = 10.3 Hz), 138.0, 133.9, 131.8, 131.2, 129.1, 128.1, 127.7, 127.4, 126.7, 122.1, 122.0, 113.7, 111.6 (d, *J* = 22.7 Hz), 109.0, 106.6, 65.3, 64.9, 55.0, 52.5, 51.3, 44.6, 40.7, 38.4, 34.7, 29.3, 24.6, 15.1, 7.5. HRMS m/z [M+H]^+^ calculated for C_39_H_41_FN_5_O_8_^+^ 726.2934, found 726.2925.

The latter compound (90 mg, 0.12 mmol) was added to a stirred mixture of MeOH (20 mL), water (10 mL) and NaOH (28 mg, 0.7 mmol) at room temperature. After stirring for 8 h, MeOH was evaporated from the mixture, the residue was cooled to 0 °C and acidified to pH 1 with conc. HCl followed by extraction with EtOAc (3 × 30 mL) to give 25 mg of crude product, which was purified by crystallization from DCM-Et_2_O to give 10 mg, 12% of pure title compound. ^1^H NMR (400 MHz, DMSO-*d*_6_) δ 15.18 (s, 1H), 9.97 (s, 1H), 8.68 (s, 1H), 8.07 (t, *J* = 5.6 Hz, 1H), 7.99–7.90 (m, 2H), 7.57 (d, *J* = 7.4 Hz, 1H), 7.48–7.35 (m, 2H), 7.25–7.19 (m, 1H), 7.12–7.05 (m, 2H), 6.86–6.80 (m, 2H), 5.18 (d, *J* = 2.9 Hz, 1H), 4.04 (d, *J* = 3.0 Hz, 1H), 3.87–3.78 (m, 1H), 3.76–3.71 (m, 1H), 3.70–3.63 (m, 4H), 3.58 (s, 2H), 3.33 (s, 4H), 3.12 (q, *J* = 6.7 Hz, 2H), 2.29 (t, *J* = 7.3 Hz, 2H), 1.74–1.60 (m, 2H), 1.36–1.27 (m, 2H), 1.22–1.14 (m, 2H). ^13^C NMR (126 MHz, DMSO-*d*_6_) δ 176.4, 170.4, 169.7, 165.9, 160.3, 158.6, 153.0 (d, *J* = 249.4 Hz), 148.1, 144.9 (d, *J* = 10.2 Hz), 139.2, 133.9, 131.8, 131.2, 129.1, 128.1, 127.7, 127.4, 126.7, 118.9, 118.8, 113.7, 111.0 (d, *J* = 23.0 Hz), 106.8, 106.6, 65.3, 55.0, 52.5, 49.5, 49.2, 44.5, 40.7, 38.4, 35.9, 29.3, 24.6, 7.6. HRMS m/z [M+Na]^+^ calculated for C_38_H_38_FN_5_O_8_Na^+^ 734.2597, found 734.2596.

#### 3.4.11. 1-Cyclopropyl-6-fluoro-7-(4-(4-((3*RS*,4*RS*)-2-hydroxy-3-(2-hydroxyphenyl)-1-oxo-1,2,3,4-tetrahydroisoquinoline-4-carboxamido)butanoyl)piperazin-1-yl)-4-oxo-1,4-dihydroquinoline-3-carboxylic acid (**8b**)

Compound **7b** (489 mg, 0.55 mmol) was placed in a round-bottom flask equipped with a magnetic stir bar and a dropping funnel capped with a calcium chloride filled drying tube followed by the addition of 100 mL of dry DCM. After cooling to 0 °C a solution of BBr_3_ (635 mg, 2.54 mmol) in DCM (50 mL) was added dropwise to the flask within 30 min. After stirring for four days at room temperature the reaction mixture was poured onto 100 mL of crushed ice and stirred for 2h. The resulting solid was filtered, washed with water (10 mL) and THF (5 mL) to give pure title compound. Yield 23 mg, 15%. ^1^H NMR (400 MHz, DMSO-*d*_6_) δ 15.19 (s, 1H), 9.99 (s, 1H), 9.88 (s, 1H), 8.67 (s, 1H), 8.00–7.90 (m, 2H), 7.83 (t, *J* = 5.6 Hz, 1H), 7.57 (d, *J* = 7.4 Hz, 1H), 7.48–7.36 (m, 2H), 7.36–7.31 (m, 1H), 7.10–7.00 (m, 1H), 6.85 (d, *J* = 8.0 Hz, 1H), 6.63–6.57 (m, 2H), 5.49 (d, *J* = 1.4 Hz, 1H), 3.95 (d, *J* = 1.5 Hz, 1H), 3.87–3.77 (m, 1H), 3.74–3.61 (m, 2H), 3.58–3.43 (m, 3H), 3.29 (s, 3H), 3.18–3.05 (m, 2H), 2.31–2.21 (m, 2H), 1.74–1.57 (m, 2H), 1.30 (d, *J* = 7.2 Hz, 2H), 1.22–1.12 (m, 2H). ^13^C NMR (126 MHz, DMSO-*d*_6_) δ 176.4, 176.4, 170.3, 170.3, 165.9, 159.6, 154.1, 152.9 (d, *J* = 249.4 Hz), 148.1, 144.9 (d, *J* = 10.2 Hz), 139.2, 133.8, 131.7, 128.7, 128.4 (d, *J* = 2.9 Hz), 127.7, 126.7, 125.7, 125.2, 118.8 (d, *J* = 7.7 Hz), 118.7, 115.2, 111.0 (d, *J* = 23.1 Hz), 106.8, 106.6 (d, *J* = 3.0 Hz), 60.0, 50.1, 49.4 (d, *J* = 26.6 Hz), 44.4, 40.7, 40.4, 38.2, 35.9, 29.1, 24.6, 7.6, 7.6. HRMS m/z [M+Na]^+^ calculated for C_37_H_36_FN_5_O_8_Na^+^ 720.2440, found 720.2445.

#### 3.4.12. 1-Cyclopropyl-6-fluoro-7-(4-(4-((3*SR*,4*SR*)-2-hydroxy-3-(2-methoxyphenyl)-1-oxo-1,2,3,4-tetrahydroisoquinoline-4-carboxamido)butanoyl)piperazin-1-yl)-4-oxo-1,4-dihydroquinoline-3-carboxylic acid (**8c**)

In a round bottom flask equipped with a rubber septum and magnetic stir bar, compound **7b** (100 mg, 0.11 mmol) was dissolved in THF/MeOH mixture (10/30 mL) followed by the addition of Pd/C (10% wt) catalyst (60 mg, 0.5 eq). Hydrogen gas (1 atm) was slowly bubbled through reaction mixture via needle for 4h and then the hydrogen inlet was removed and the reaction mixture was left stirring under atmosphere of hydrogen overnight. The reaction progress was monitored via HPLC. After completion of the reaction the reaction mixture was filtered through a pad of Celite, which was washed with THF (20 mL) and ACN (20 mL). This filtrate contained admixture only and was discarded. Celite was further washed with hot DMF (20 mL) to give 65 mg of crude product, which was purified by precipitation from DCM solution (10 mL) with Et_2_O to give **8c**. Yield 30 mg, 38%. ^1^H NMR (400 MHz, DMSO-*d*_6_) δ 15.18 (s, 1H), 10.02 (s, 1H), 8.67 (s, 1H), 8.00–7.89 (m, 2H), 7.84 (t, *J* = 5.7 Hz, 1H), 7.57 (d, *J* = 7.4 Hz, 1H), 7.47–7.36 (m, 2H), 7.29 (d, *J* = 7.2 Hz, 1H), 7.22 (t, *J* = 7.7 Hz, 1H), 7.04 (d, *J* = 8.2 Hz, 1H), 6.77 (t, *J* = 7.5 Hz, 1H), 6.68 (d, *J* = 7.6 Hz, 1H), 5.49 (s, 1H), 3.91 (s, 1H), 3.88 (s, 3H), 3.82 (s, 1H), 3.67 (s, 3H), 3.53 (s, 2H), 3.30 (s, 3H), 3.12 (q, *J* = 6.5 Hz, 2H), 2.28 (t, *J* = 7.3 Hz, 2H), 1.75–1.61 (m, 2H), 1.31 (d, *J* = 6.9 Hz, 2H), 1.21–1.13 (m, 2H). ^13^C NMR (126 MHz, DMSO-*d*_6_) δ 176.3, 170.4, 170.2, 165.9, 159.7, 156.0, 152.9 (d, *J* = 249.4 Hz), 148.0, 144.9 (d, *J* = 9.9 Hz), 139.1, 133.7, 131.8, 128.9, 128.7, 128.4, 127.7, 126.8, 126.7, 125.6, 120.1, 118.8 (d, *J* = 8.0 Hz), 111.2, 111.0 (d, *J* = 23.1 Hz), 106.8, 106.6, 60.1, 55.7, 50.3, 49.5, 49.2, 44.5, 40.7, 38.3, 35.9, 29.2, 24.7, 7.6, 7.6. HRMS m/z [M+Na]^+^ calculated for C_38_H_38_FN_5_O_8_Na^+^ 734.2597, found 734.2591.

#### 3.4.13. 1-Cyclopropyl-6-fluoro-7-(4-(4-((3S,4S)-3-(2-fluorophenyl)-2-hydroxy-1-oxo-1,2,3,4-tetrahydroisoquinoline-4-carboxamido)butanoyl)piperazin-1-yl)-4-oxo-1,4-dihydroquinoline-3-carboxylic acid (**8d**)

In a round bottom flask equipped with a rubber septum and magnetic stir bar, compound **7c** (53 mg, 0.07 mmol) was dissolved in MeOH (20 mL) followed by the addition of Pd/C (10% wt) catalyst (50 mg, 0.7 eq). Hydrogen gas (1 atm) was slowly bubbled through the reaction mixture via needle for 4h and then the hydrogen inlet was removed and the reaction mixture was left stirring under atmosphere of hydrogen overnight. The reaction progress was monitored via HPLC. After completion of the reaction the reaction mixture was filtered through a pad of Celite, which was washed with THF (20 mL). The filtrate was concentrated and the residue was purified by preparative RP-HPLC (ACN-water + 0.1% TFA; gradient 20–90% of ACN in 35 min; 40 °C, 12 mL/min) to give 18 mg, 41% of pure title compound as yellow oil. ^1^H NMR (400 MHz, DMSO-*d*_6_) δ 8.67 (s, 1H), 8.02–7.96 (m, 1H), 7.96–7.89 (m, 2H), 7.57 (d, *J* = 7.4 Hz, 1H), 7.53–7.40 (m, 2H), 7.37–7.27 (m, 2H), 7.27–7.18 (m, 1H), 7.13–7.04 (m, 1H), 6.95–6.86 (m, 1H), 5.51 (d, *J* = 2.9 Hz, 1H), 4.07 (d, *J* = 3.0 Hz, 1H), 3.87–3.78 (m, 1H), 3.67 (d, *J* = 5.4 Hz, 2H), 3.55 (s, 2H), 3.30 (s, 4H), 3.11 (q, *J* = 6.4 Hz, 2H), 2.25 (t, *J* = 7.3 Hz, 2H), 1.72–1.54 (m, *J* = 6.8 Hz, 2H), 1.36–1.27 (m, 2H), 1.18 (d, *J* = 3.8 Hz, 2H). ^13^C NMR (100 MHz, DMSO-*d*_6_) δ176.4 (d, *J* = 2.4 Hz), 170.3, 169.5, 165.9, 160.3, 159.6 (d, *J* = 245.3 Hz), 153.0 (d, *J* = 249.3 Hz), 148.1, 144.9 (d, *J* = 10.2 Hz), 139.2, 133.5, 132.2, 129.9 (d, *J* = 8.5 Hz), 128.5, 128.1, 128.0, 127.4, 127.4, 127.0, 126.1 (d, *J* = 12.9 Hz), 124.4 (d, *J* = 3.0 Hz), 118.8 (d, *J* = 7.6 Hz), 115.7 (d, *J* = 21.3 Hz), 111.0 (d, *J* = 22.8 Hz), 106.8, 106.6 (d, *J* = 3.2 Hz), 59.7 (d, *J* = 2.9 Hz), 50.9, 49.5, 49.2, 44.5, 40.7, 38.4, 35.9, 29.2, 24.5, 7.6. HRMS m/z [M+Na]^+^ calculated for C_37_H_35_F_2_N_5_O_7_Na^+^ 722.2397, found 722.2393.

## 4. Conclusions

The screening of a library of novel *N*-hydroxylactams amenable by the Castagnoli-Cushman reaction identified four lead compounds that exerted a minimal effect on the growth of *P. aeruginosa ∆pvdF∆pchA* mutant strain grown in (iron-restricted) CAA medium and transported ^55^Fe into *P. aeruginosa* cells. Notably, one of these synthetic siderophores was found to be as efficient at promoting iron uptake as the natural siderophores pyoverdine, pyochelin or enterobactin. Conjugates of the four lead siderophores with ciprofloxacin were synthesized via sequential amide coupling and protecting group manipulation. These were tested for antibacterial activity against *P. aeruginosa* POA1 (wild type) and the *∆pvdF∆pchA* mutant strain. For bacteria grown under non-restricted iron conditions (in Müller-Hinton medium), no antibacterial activity was observed. Likewise, none of the siderophore-ciprofloxacin constructs was active against the POA1 strain grown in iron-restricted conditions (CAA medium). However, the antibacterial activity was found to be pronounced against the *∆pvdF∆pchA* mutant strain grown in CAA medium. This may be indicative of the uptake of these compounds by the iron-starved bacteria and of their being ‘Trojan horse’ antibiotics. Further scrutiny of the mechanism of the antibacterial action of the newly developed conjugates is warranted.

## Figures and Tables

**Table 1:** MIC values determined for ciprofloxacin-siderophore conjugates **8a–d** against *P. aeruginosa* PAO1 and *∆pvdF∆pchA* strains grown in MH or CAA (iron-restricted) medium (both strains were grown in the presence of increasing concentrations of **8a–d**; the MIC values were measured in μM after 48 h in triplicates; ciprofloxacin was used as the positive control displaying an MIC value of 48 ± 1.2 μM).

Compound	MH Medium		CAA Medium	
	PAO1	*∆pvdF∆pchA*	PAO1	*∆pvdF∆pchA*
**8a**	>64	64	>64	1
**8b**	>64	64	>64	1
**8c**	>64	64	>64	1
**8d**	>64	64	>64	1

## Data Availability

Data available from the corresponding authors upon reasonable request.

## Supplementary Materials

The following supporting information can be downloaded at: https://www.mdpi.com/article/10.3390/molecules27123910/s1. Structures of the compounds screened. Copies of NMR spectra. Bacterial growth graphs. Figure S1: Growth of ΔpvdFΔpchA P. aeruginosa mutant in the absence or presence of 10 μM and 100 μM of the different compounds in iron-restricted medium. The growth medium used was CAA and growth was followed by monitoring optical density (OD) at 600 nm at 30 °C.

## Sample Availability

Samples of the compounds **8a-d** are available from the authors.
