# Supplementary material for: Conjugates of Iron-Transporting N-Hydroxylactams with Ciprofloxacin
Source: Molecules. 2022 Jun 18;27(12):3910. doi: 10.3390/molecules27123910 (PMC9228471; doi:10.3390/molecules27123910)
Supplement: Supplementary file 1 [file molecules-27-03910-s001.zip › molecules-1768464-supplementary.pdf]

Supplementary Materials for

Conjugates of iron-transporting *N*-hydroxylactams with ciprofloxacin

Olga Bakulina, Anton Bannykh, Ekaterina Levashova and Mikhail Krasavin*

Contents

Copies of ^{1}H and ^{13}C NMR spectra	2-14
Structures of compounds **1a-z**	15-16
Bacterial growth graphs (Figure S1)	17

^{1}H and ^{13}C spectra of compound 4

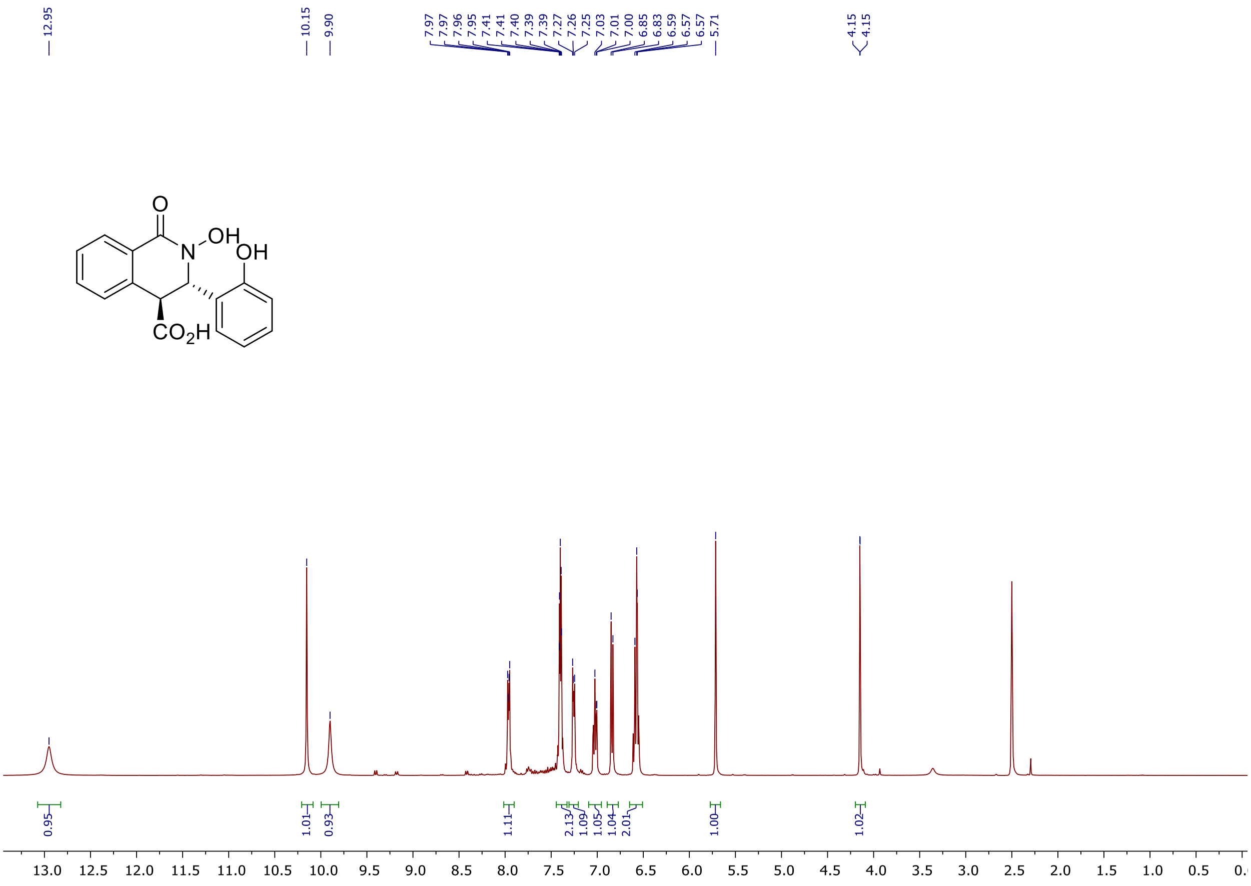

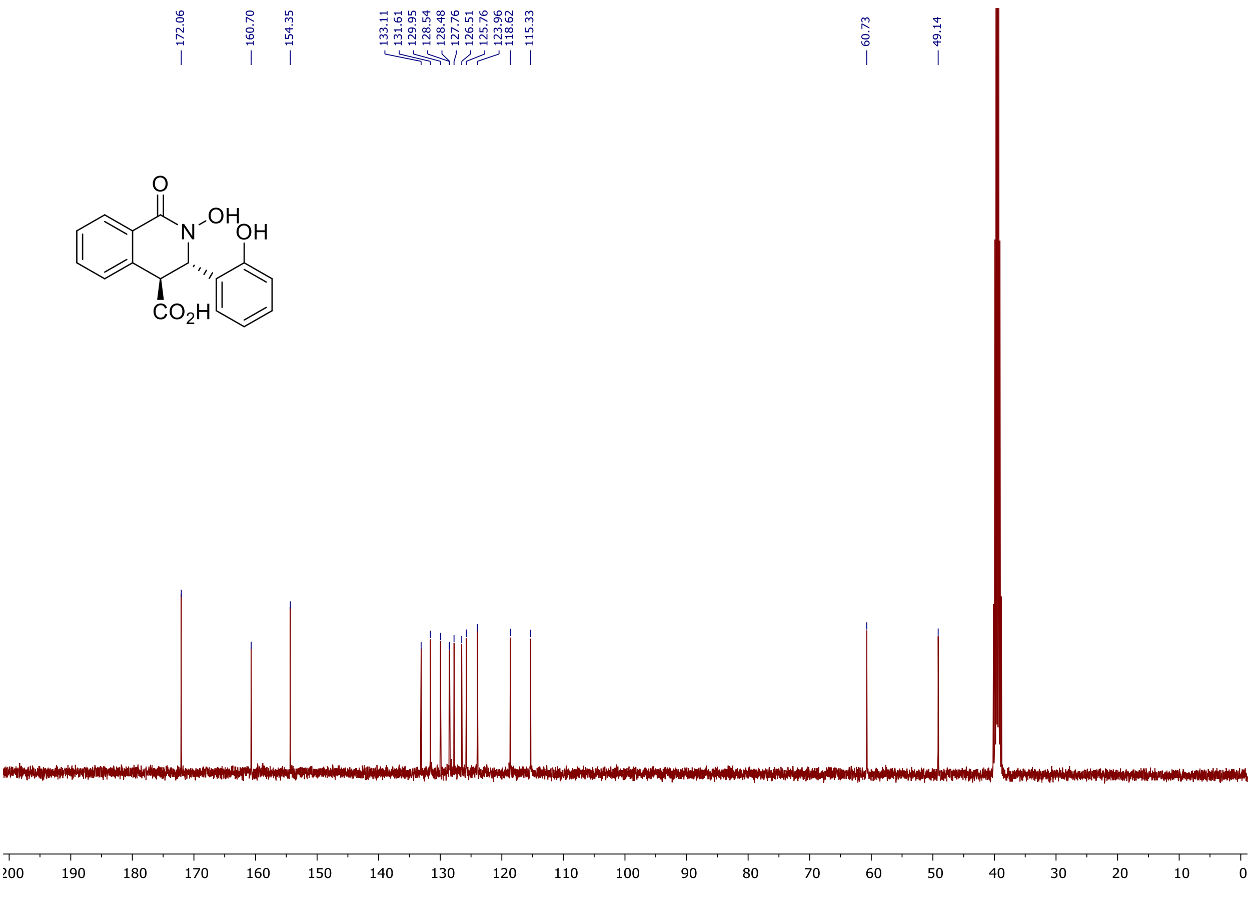

^{1}H and ^{13}C spectra of compound 5b

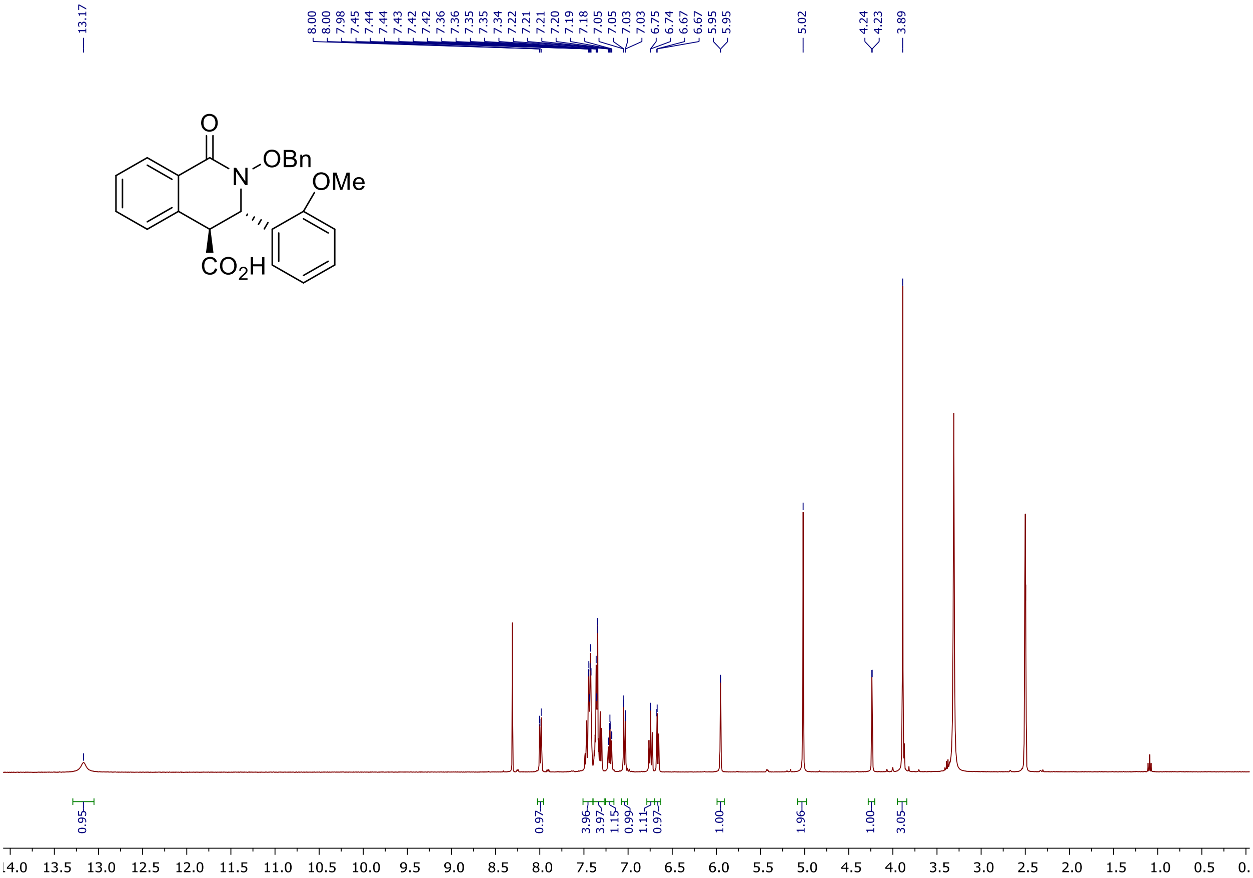

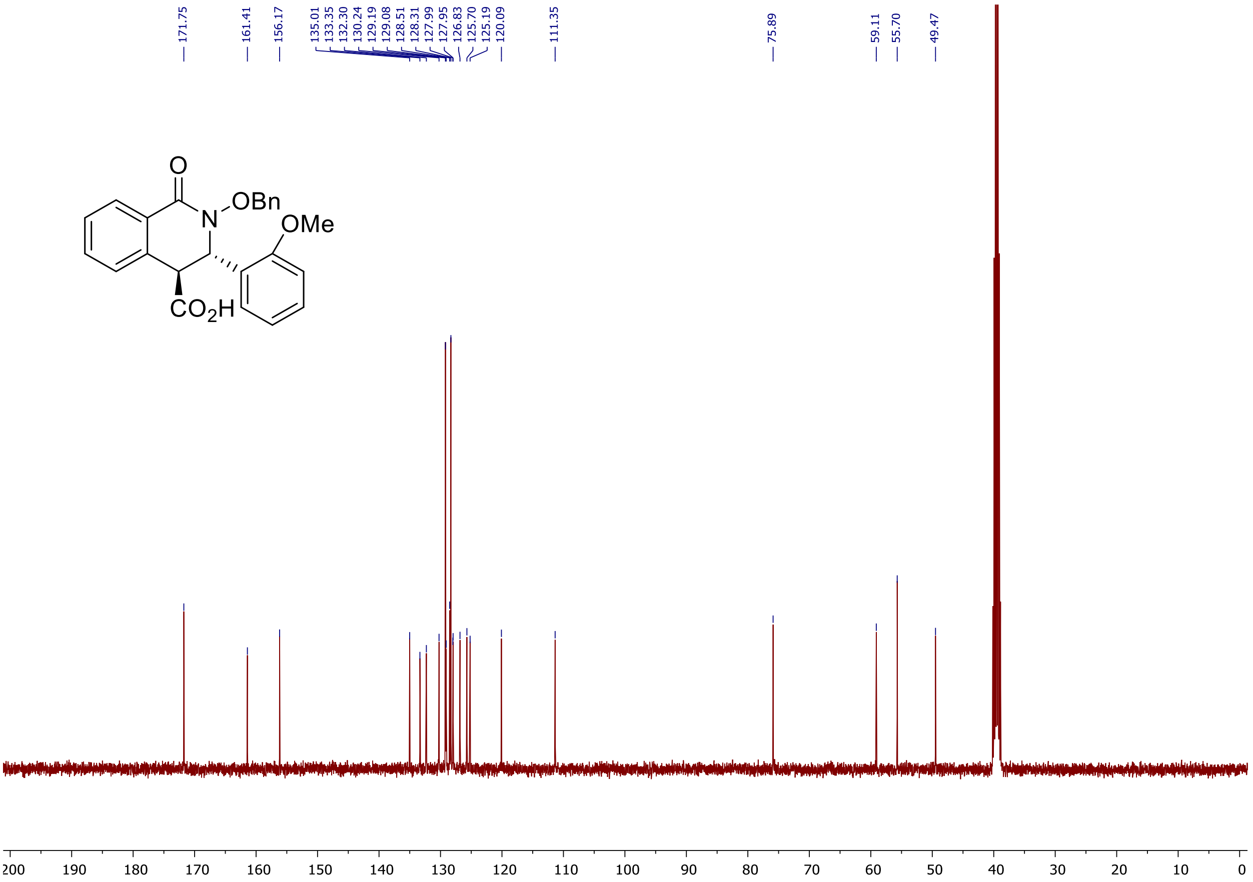

^{1}H and ^{13}C spectra of compound 5c

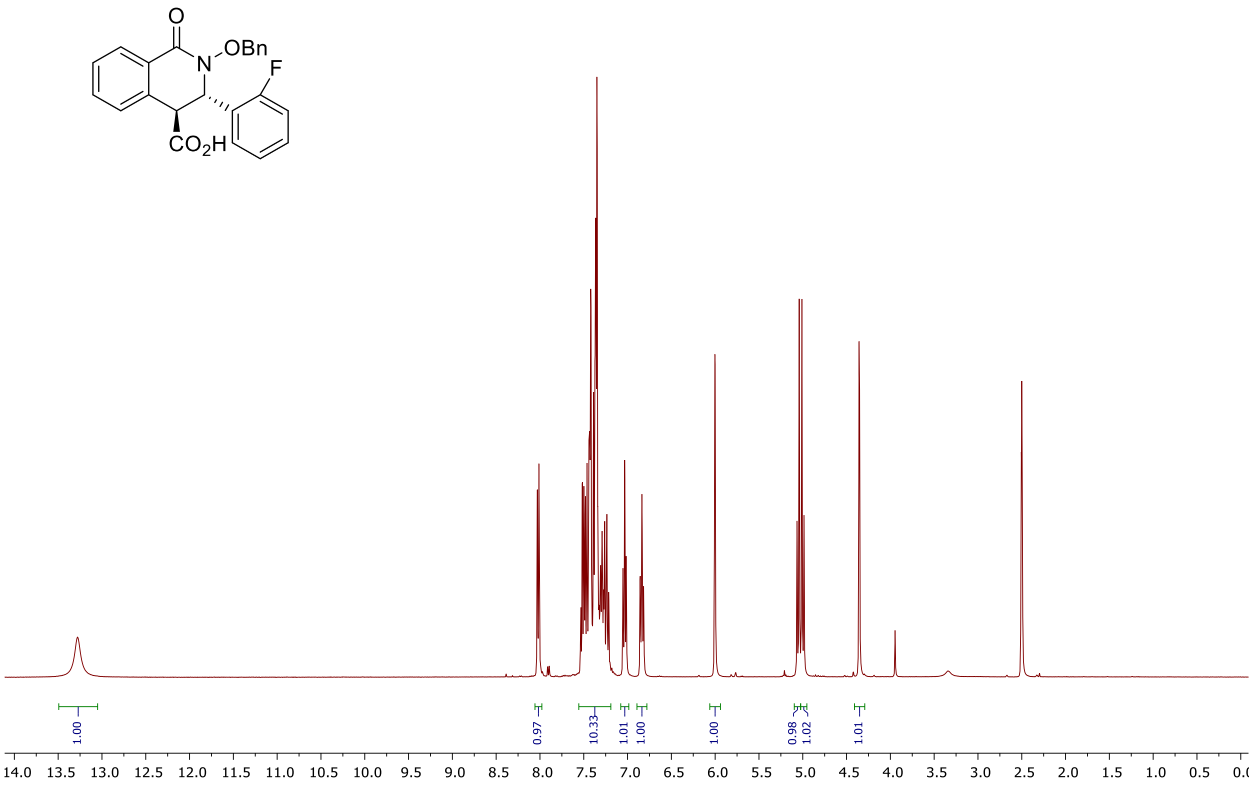

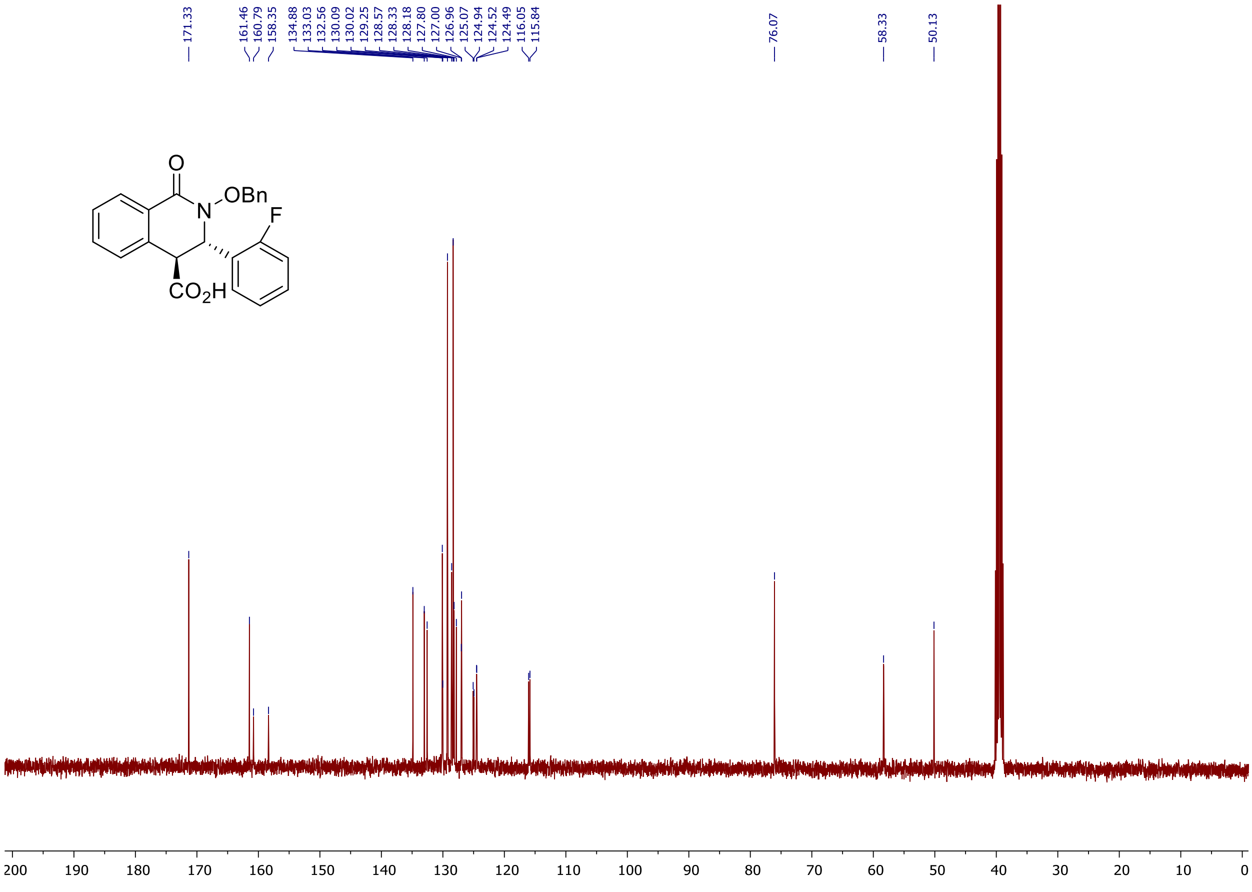

^{1}H and ^{13}C spectra of compound 6a

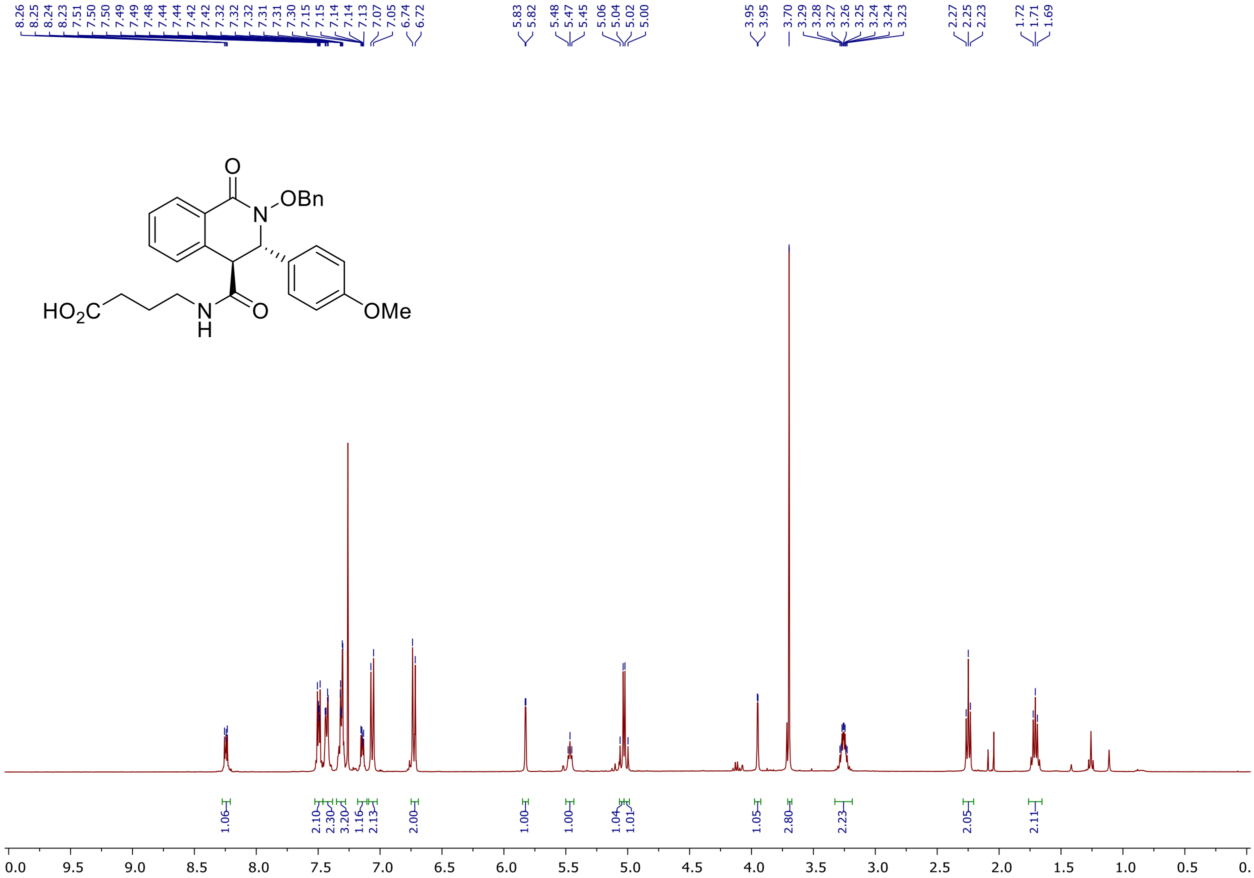

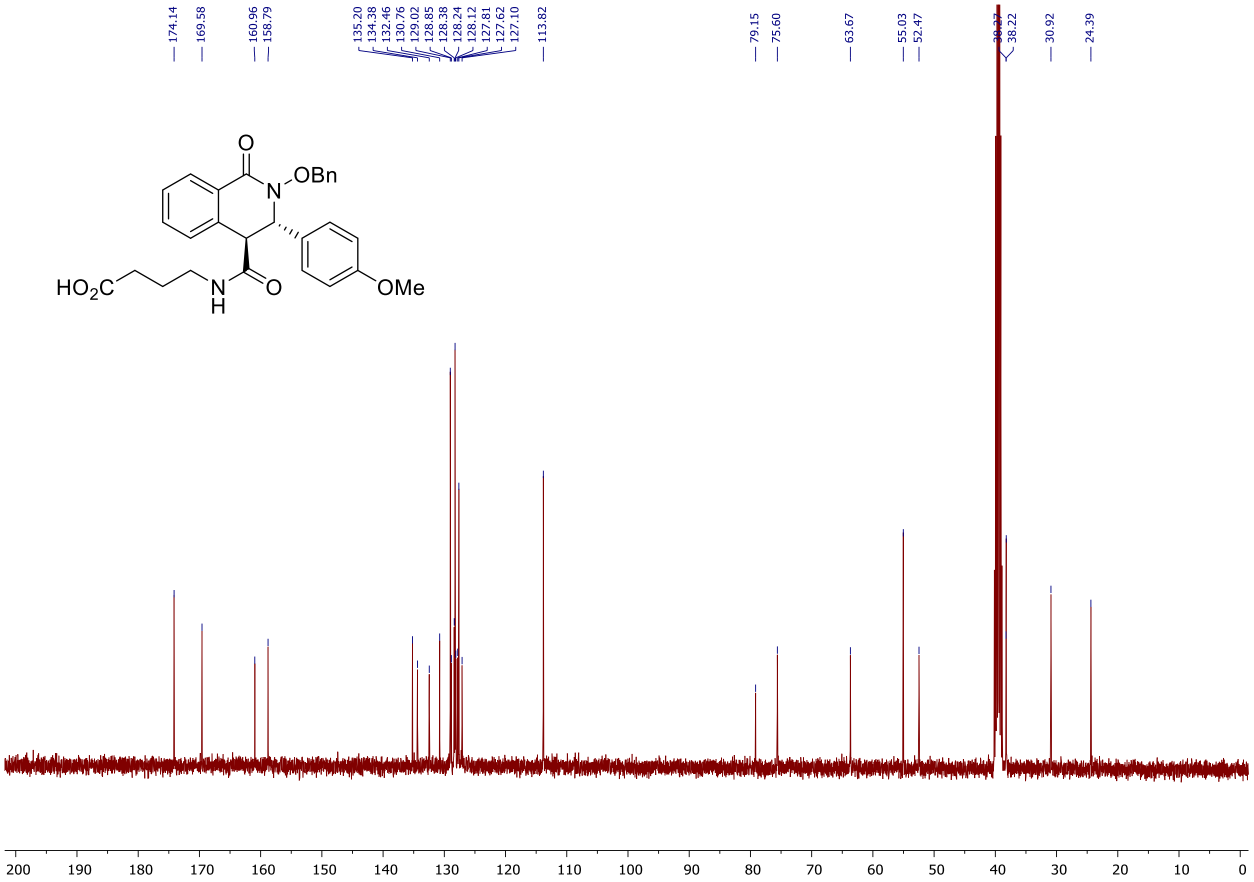

^{1}H and ^{13}C spectra of compound 6b

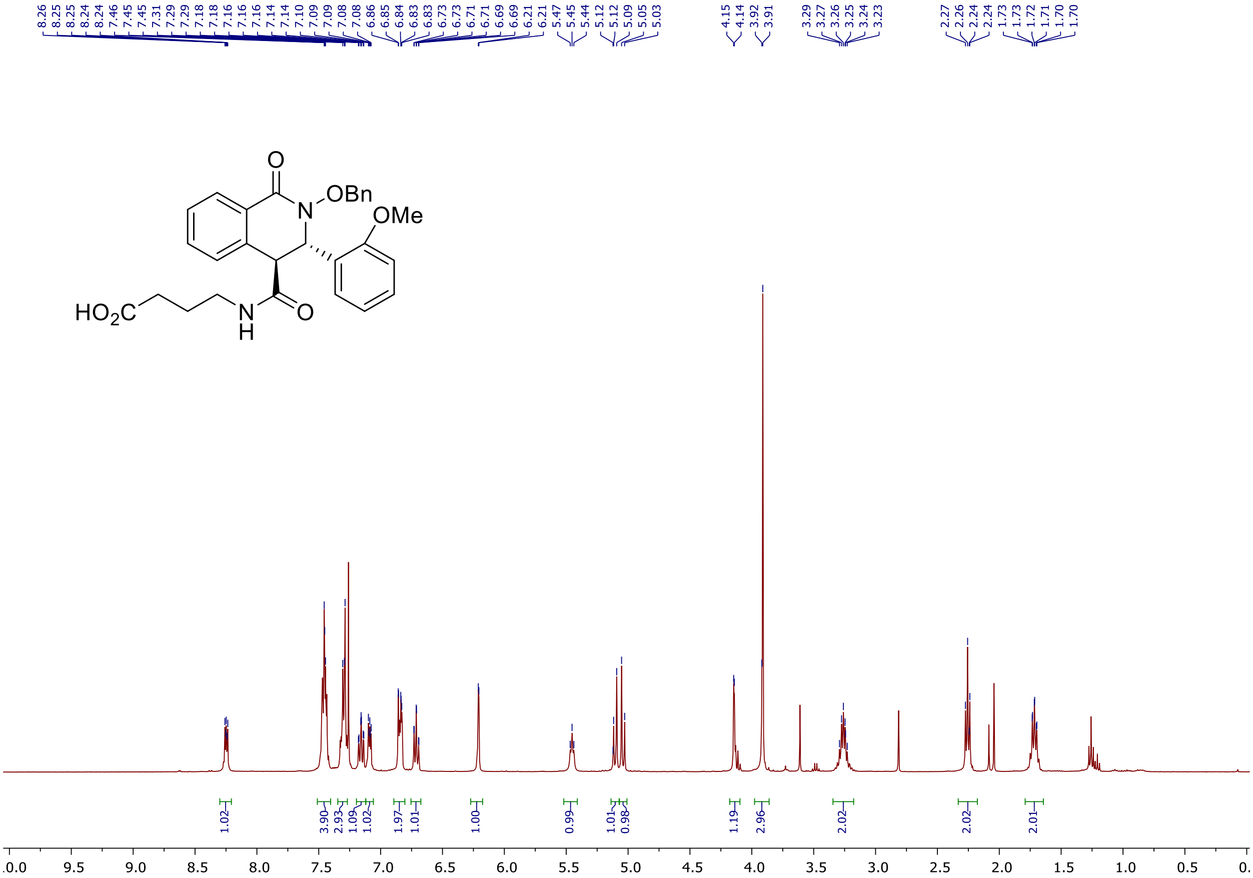

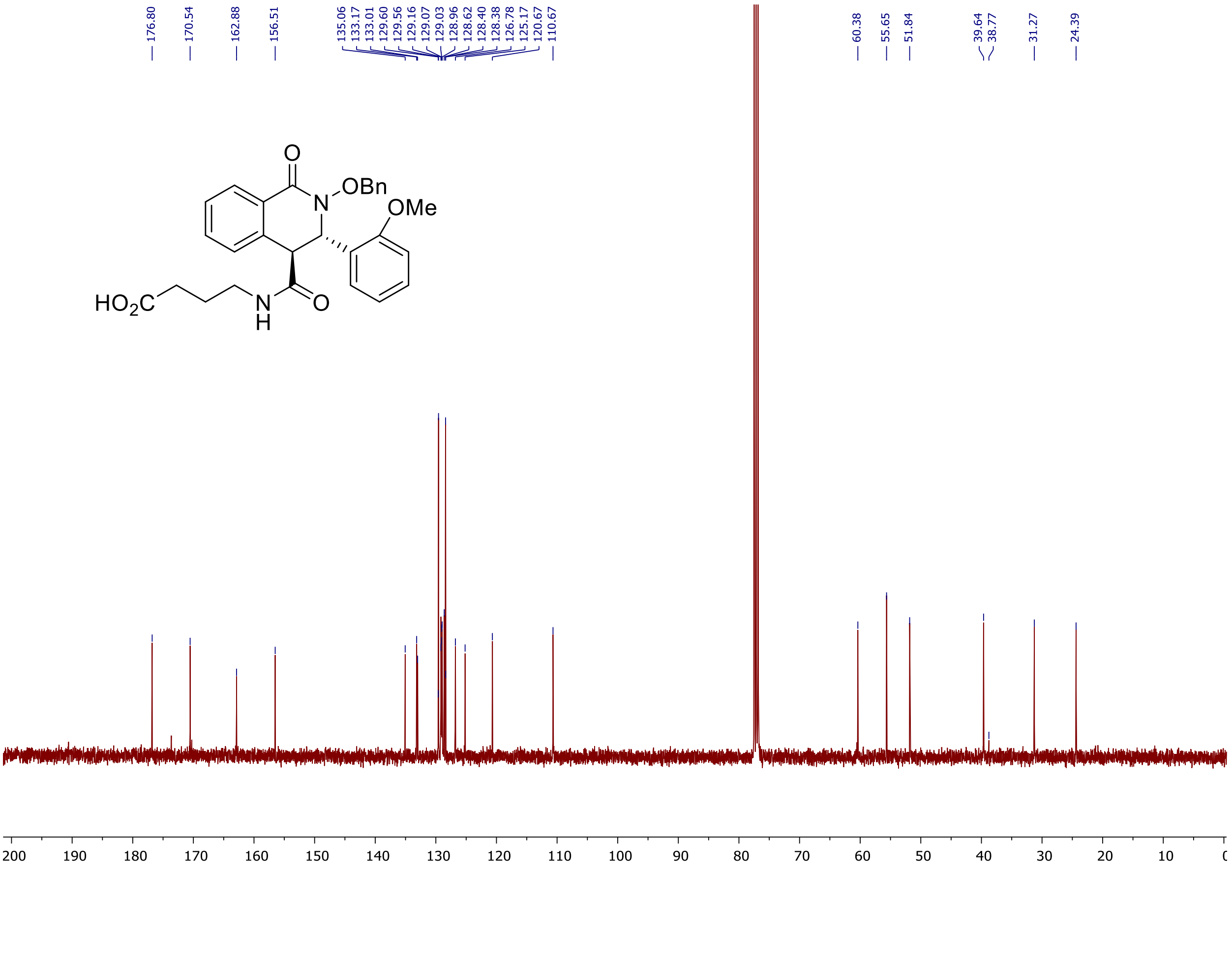

^{1}H and ^{13}C spectra of compound 6c

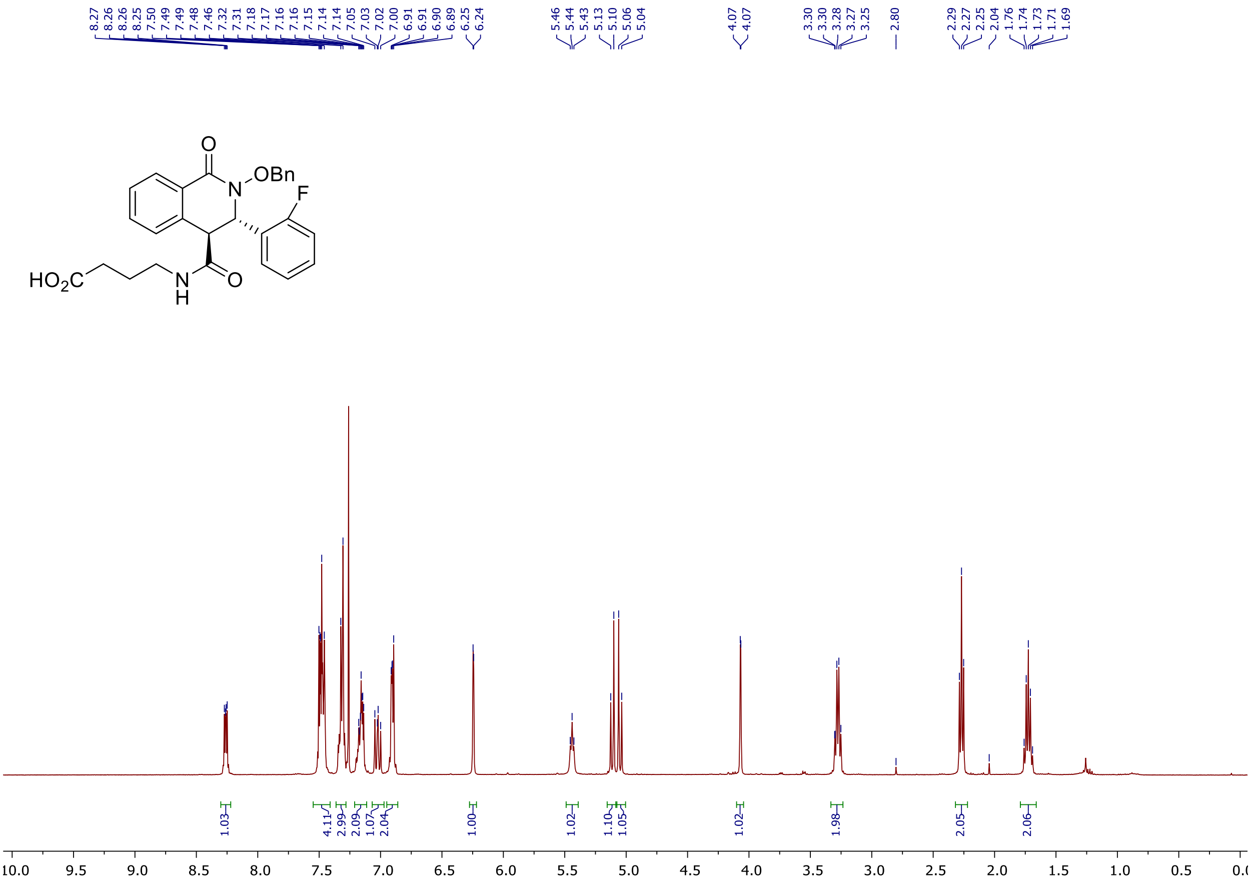

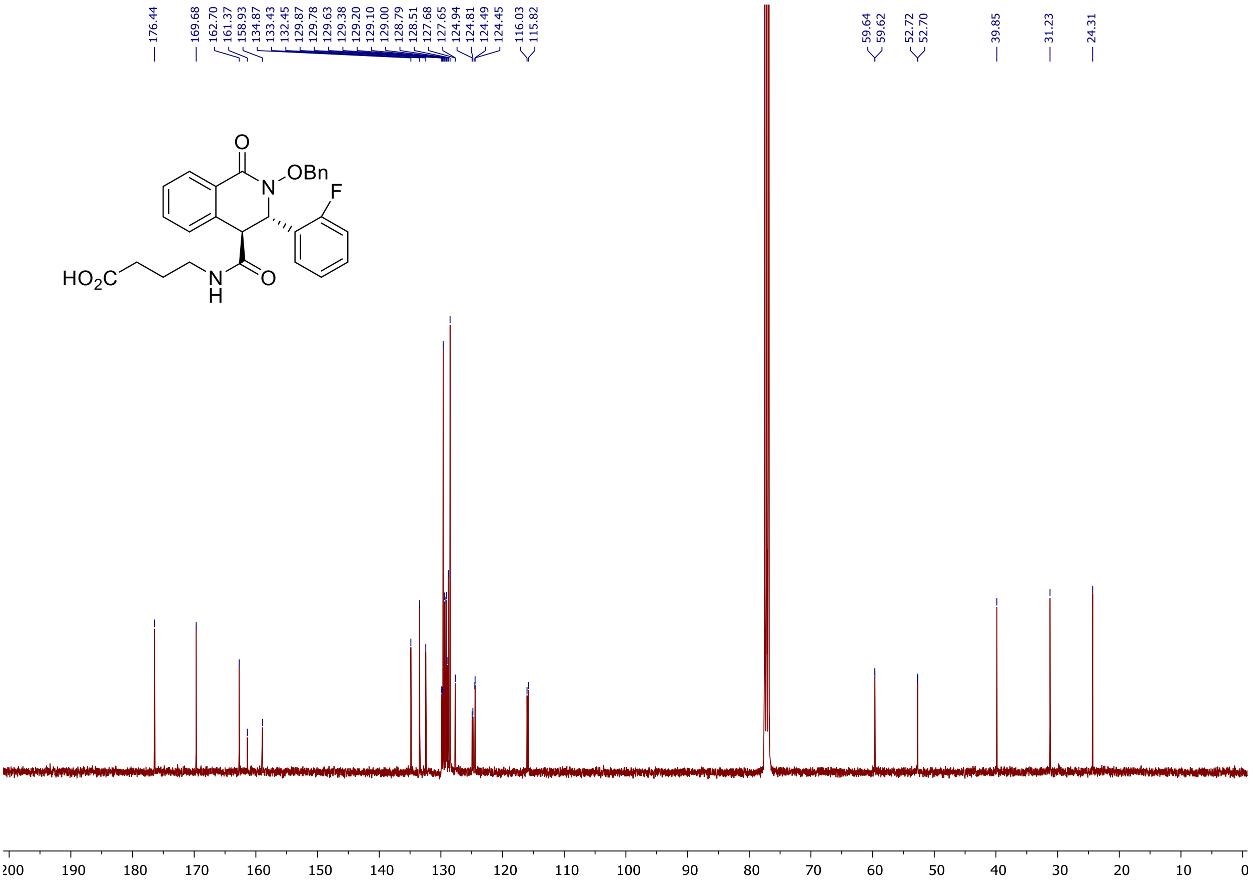

^{1}H and ^{13}C spectra of compound 7a

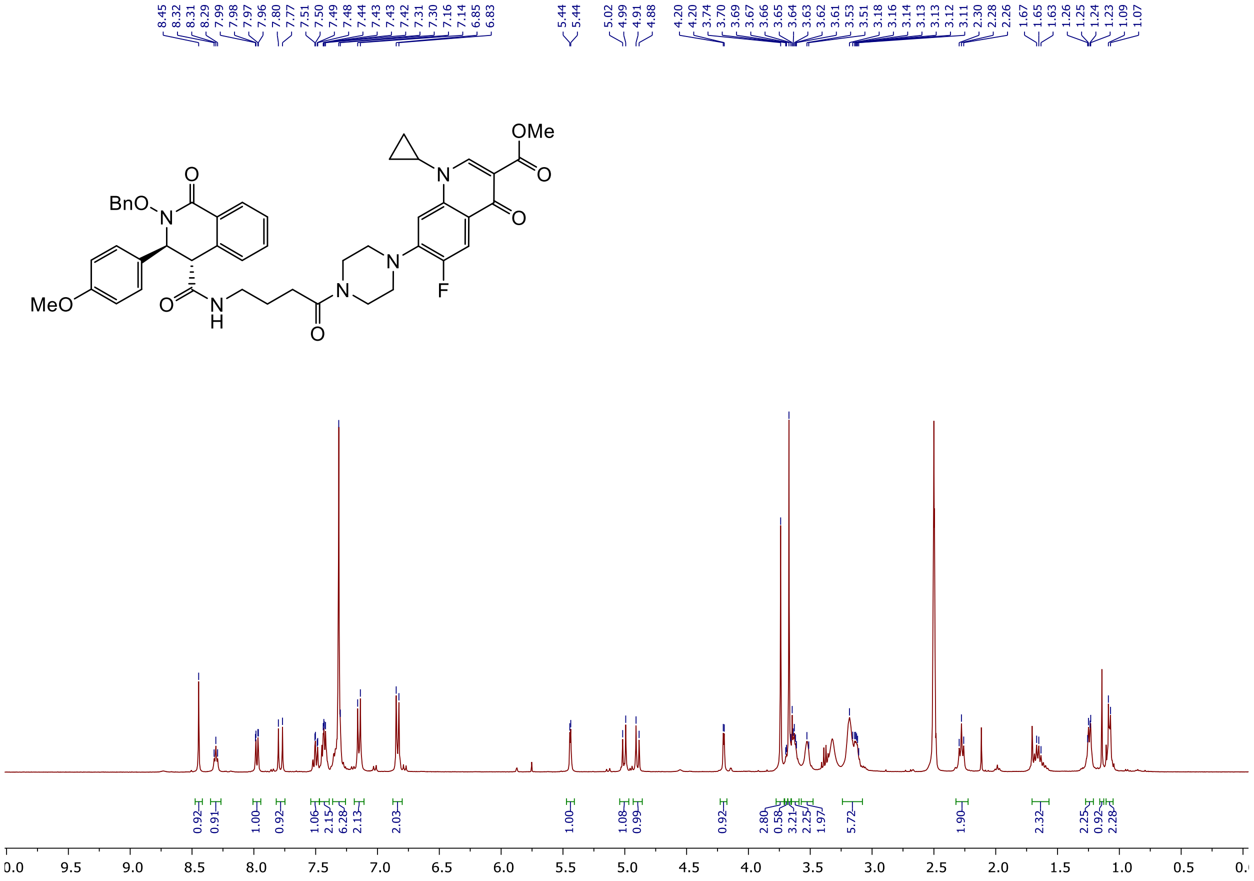

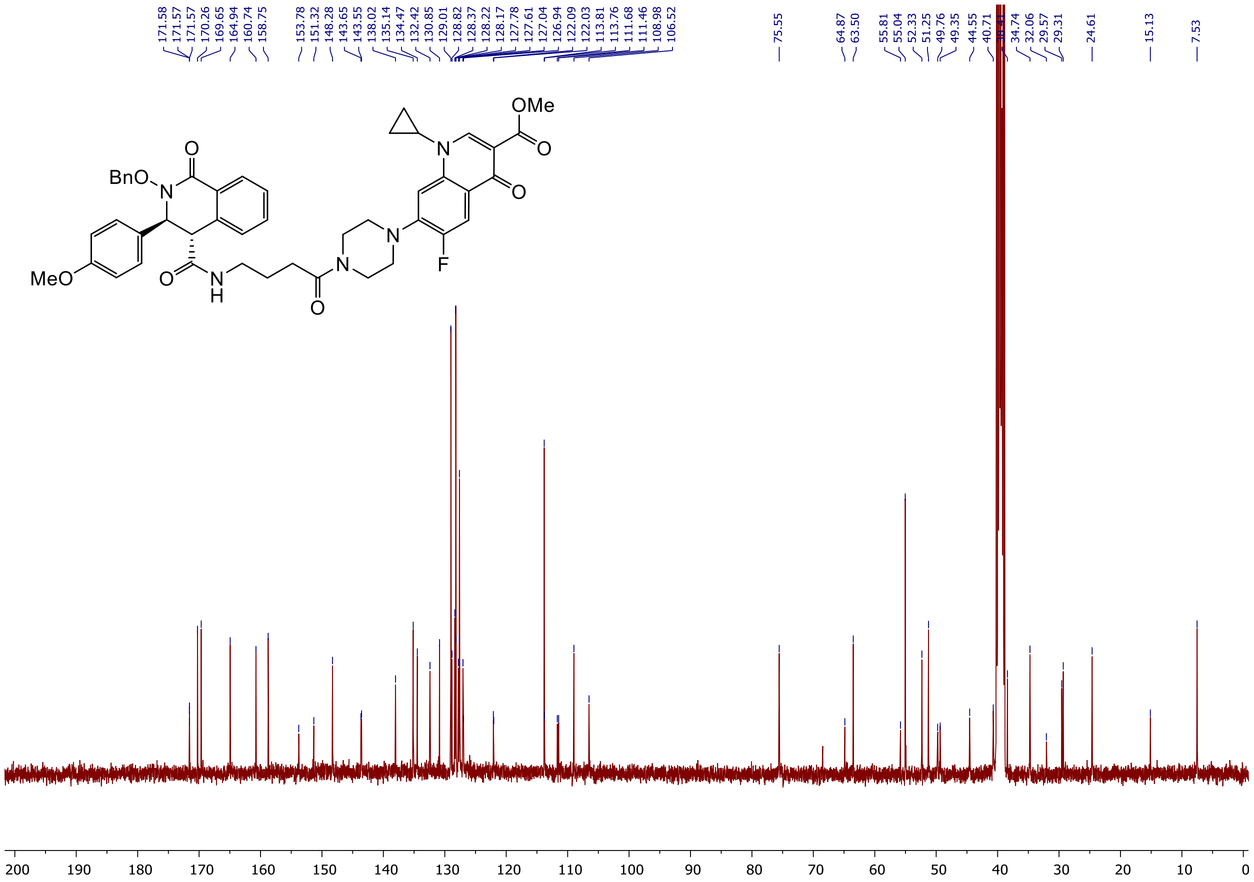

^{1}H and ^{13}C spectra of compound 7b

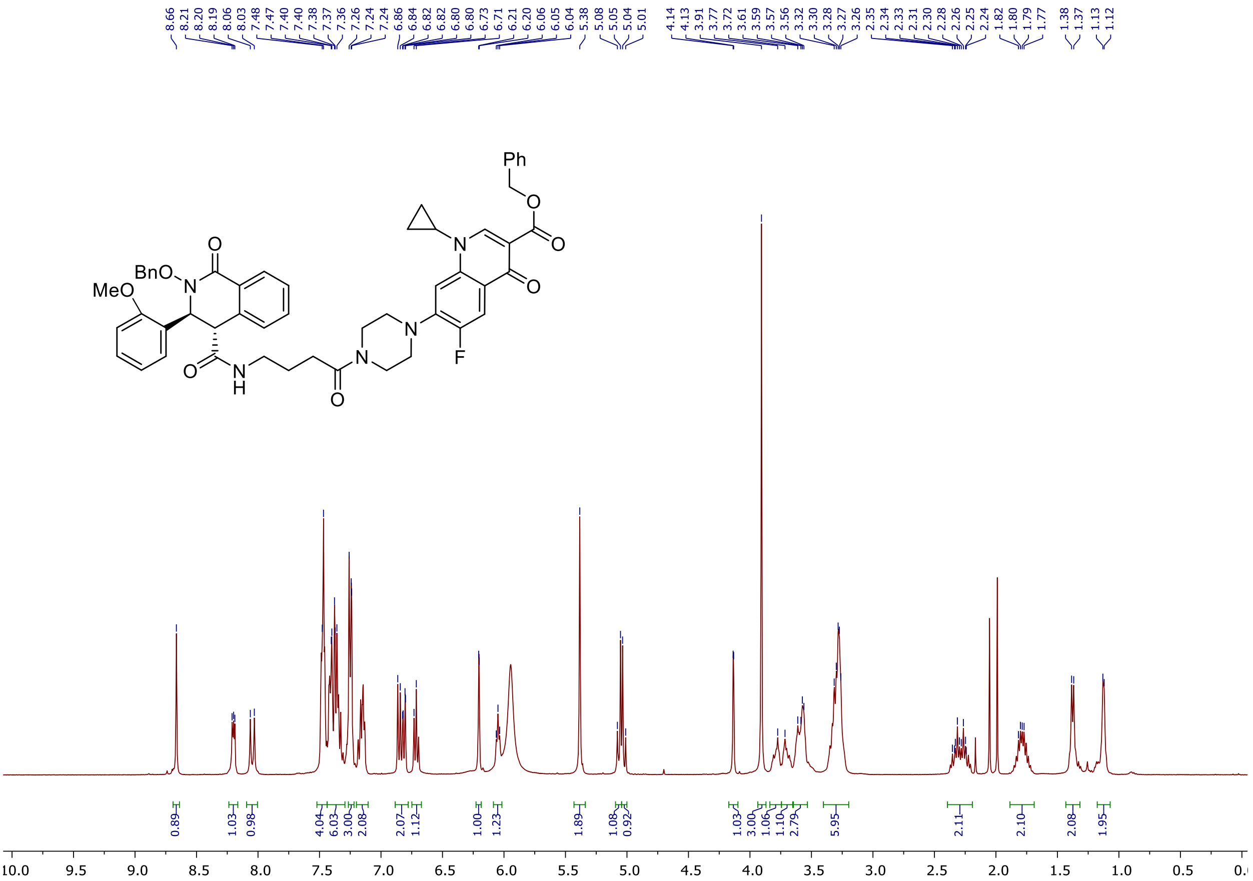

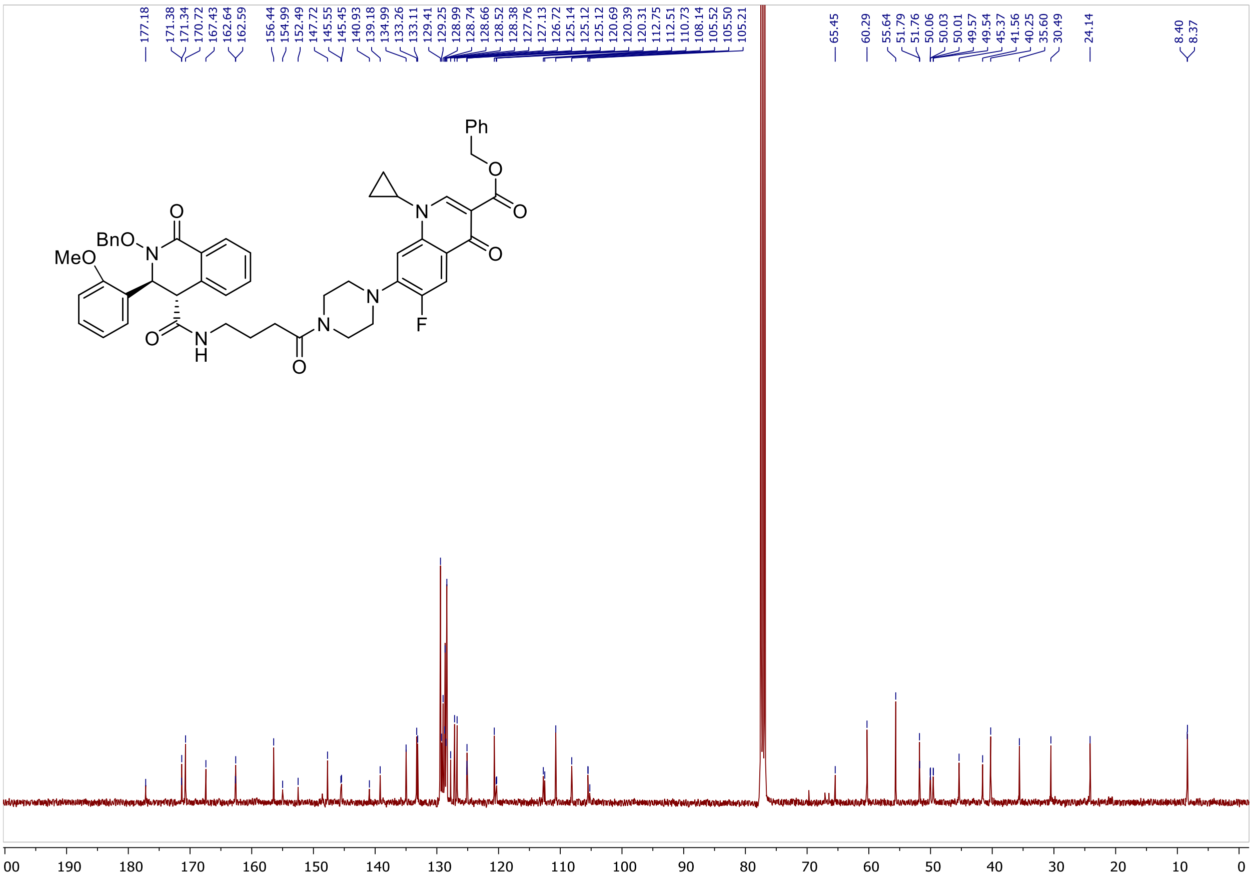

^{1}H and ^{13}C spectra of compound 7c

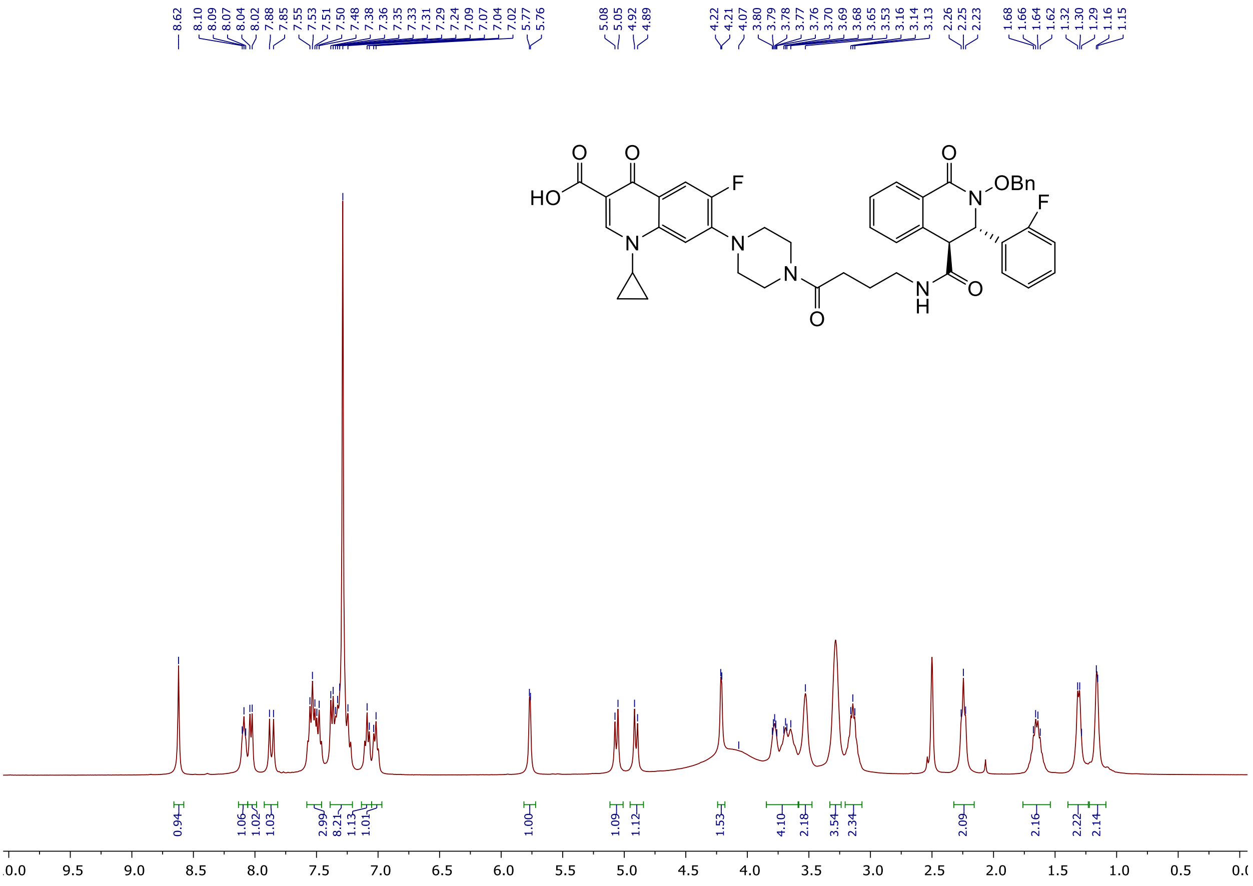

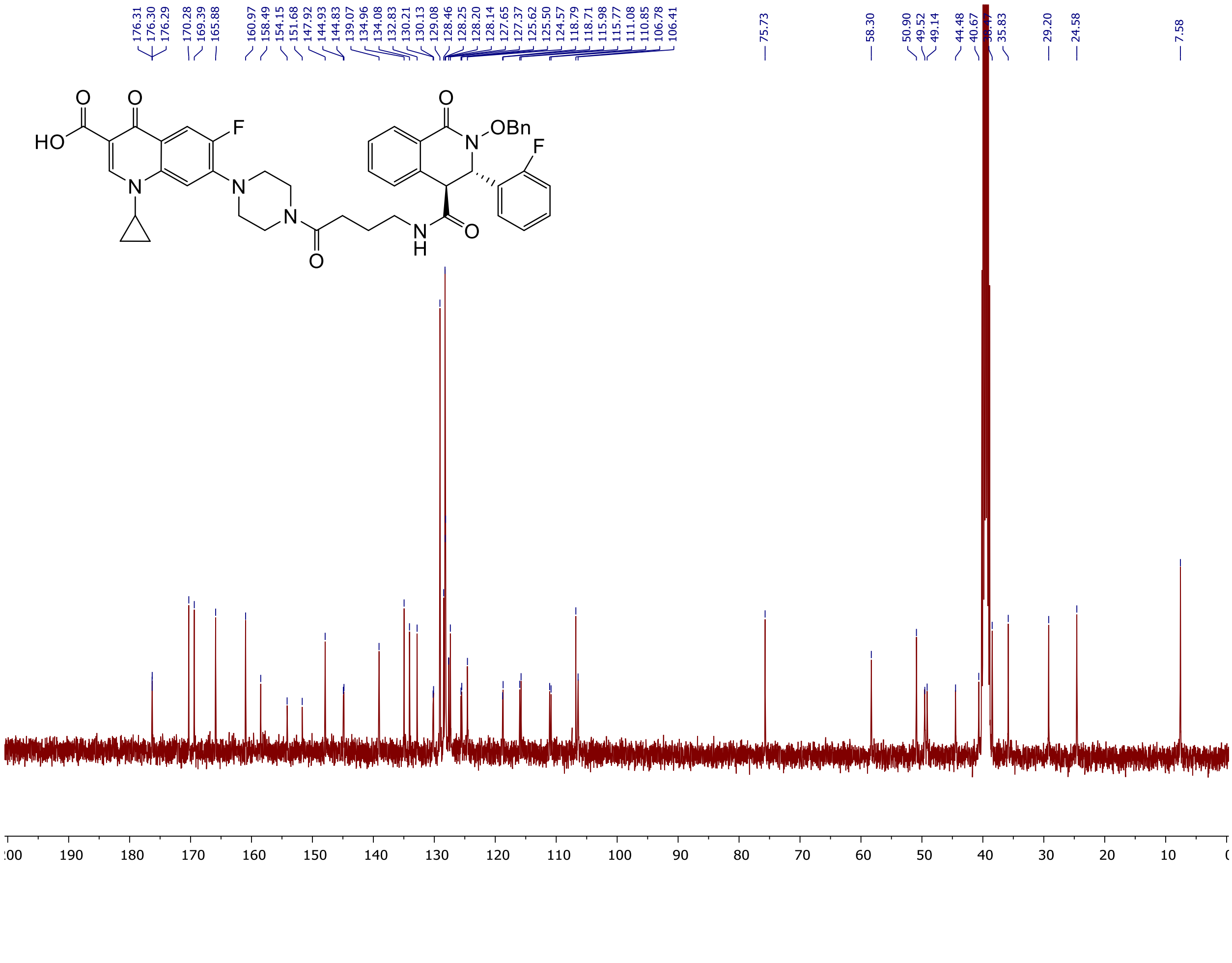

^{1}H and ^{13}C spectra of compound 8a

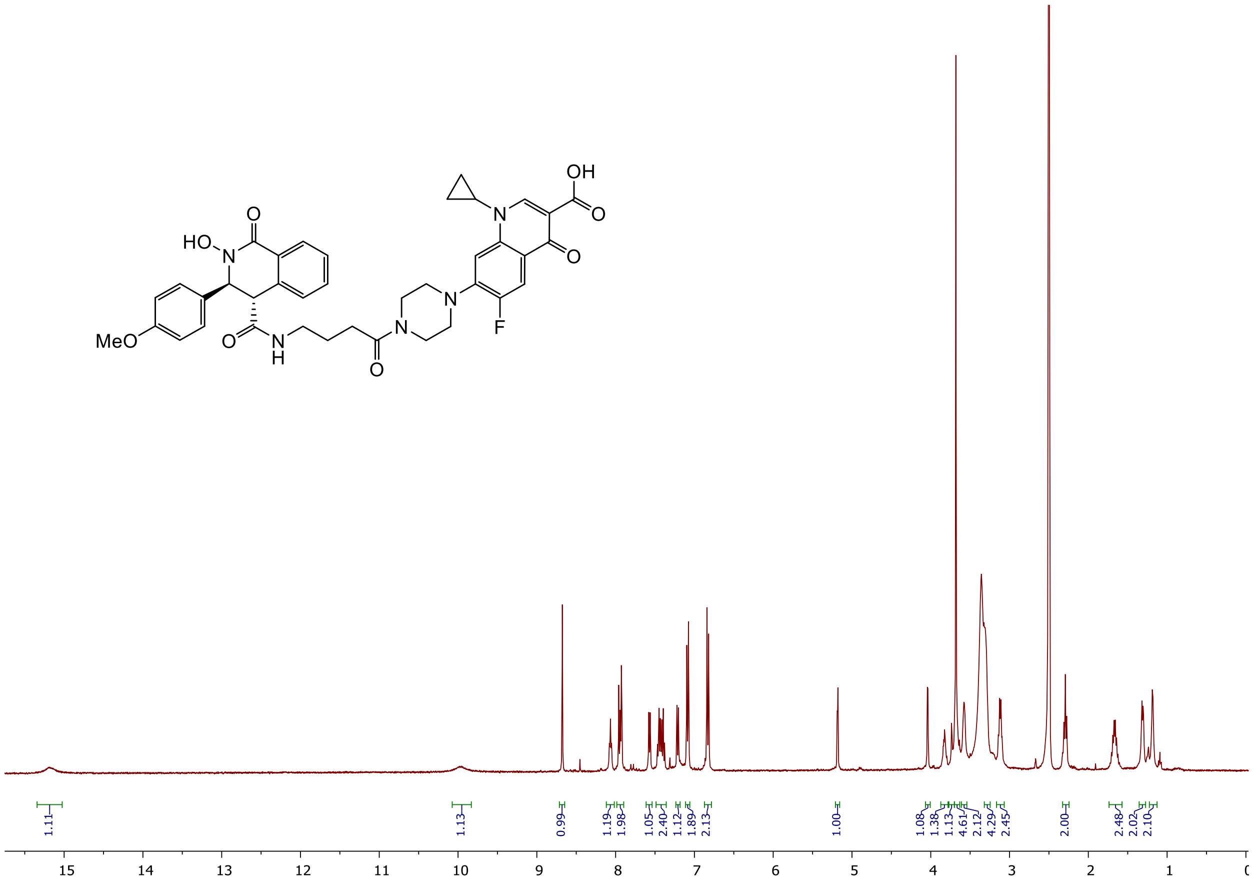

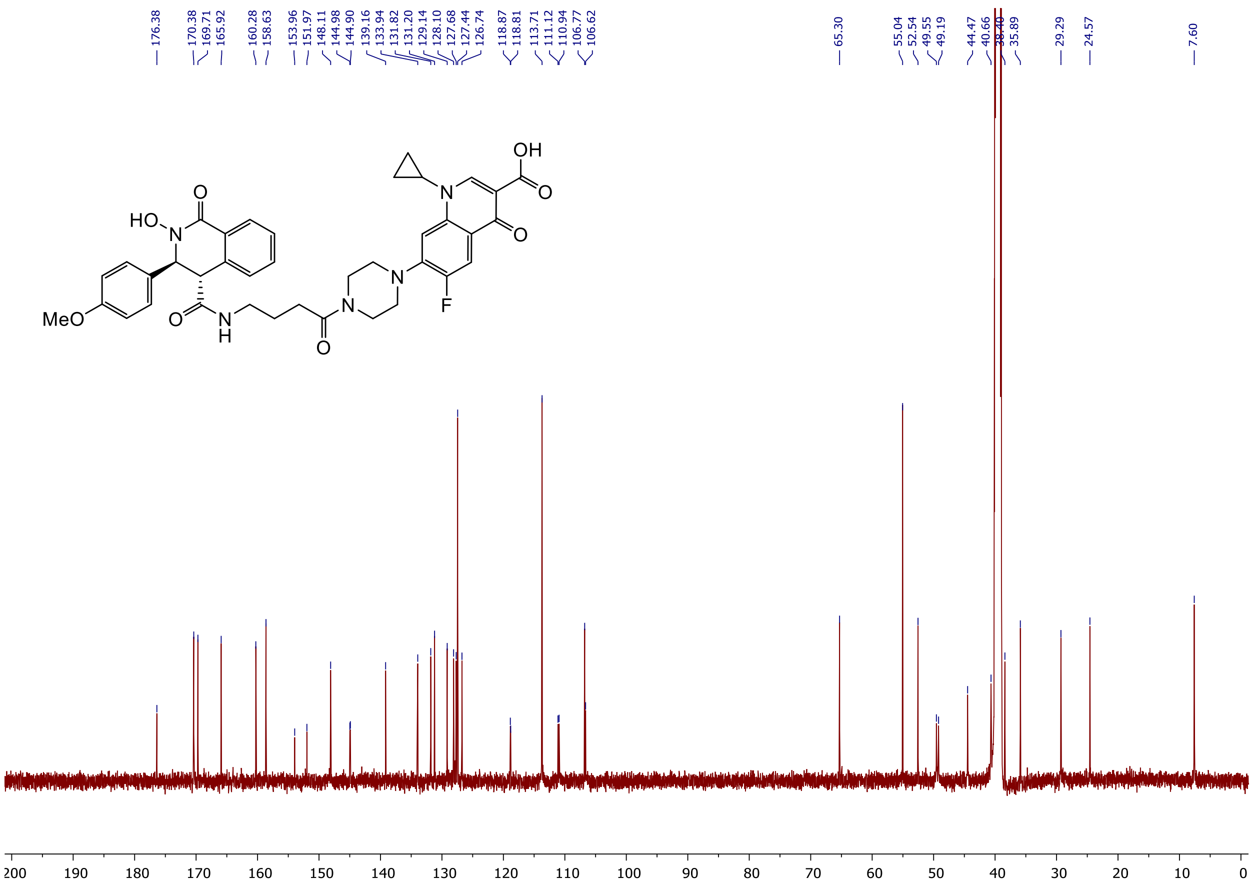

^{1}H and ^{13}C spectra of compound 8b

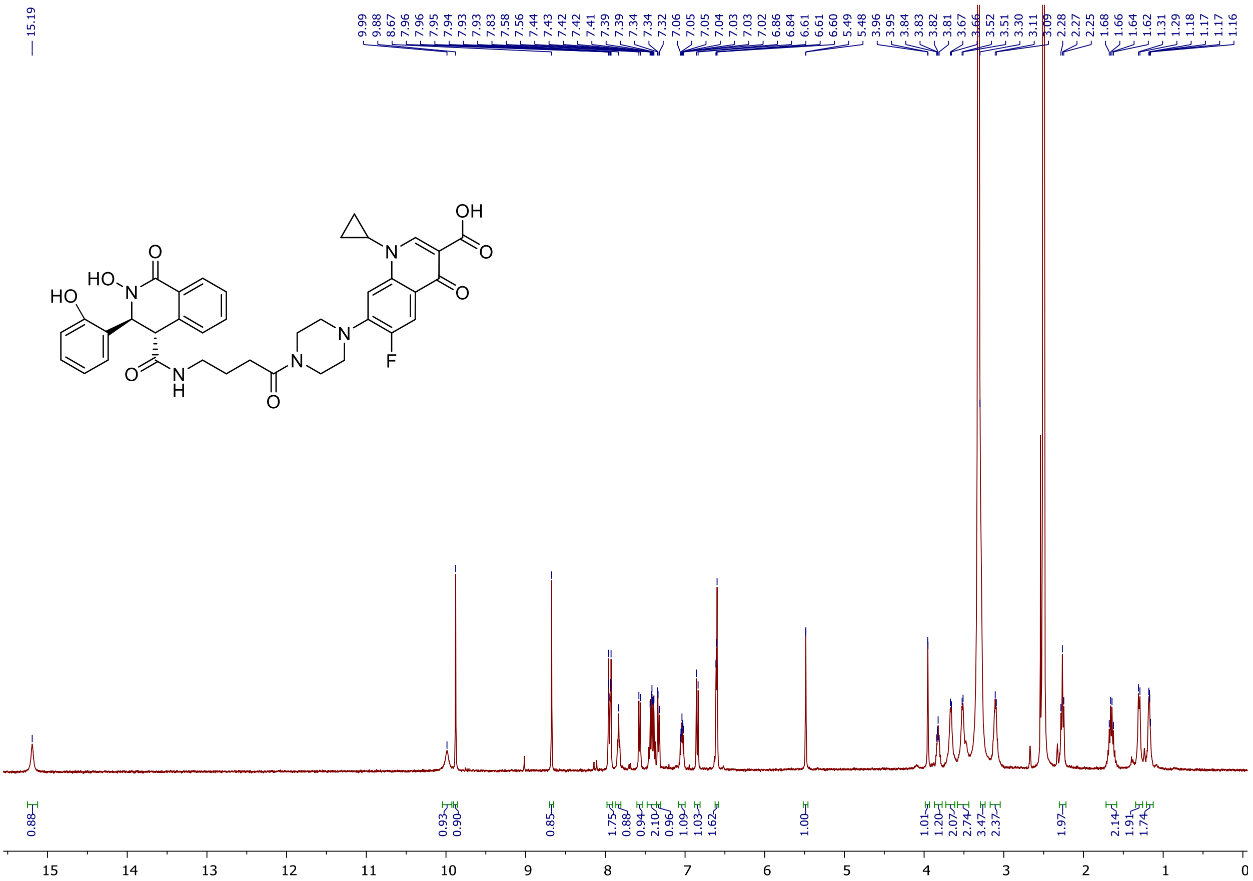

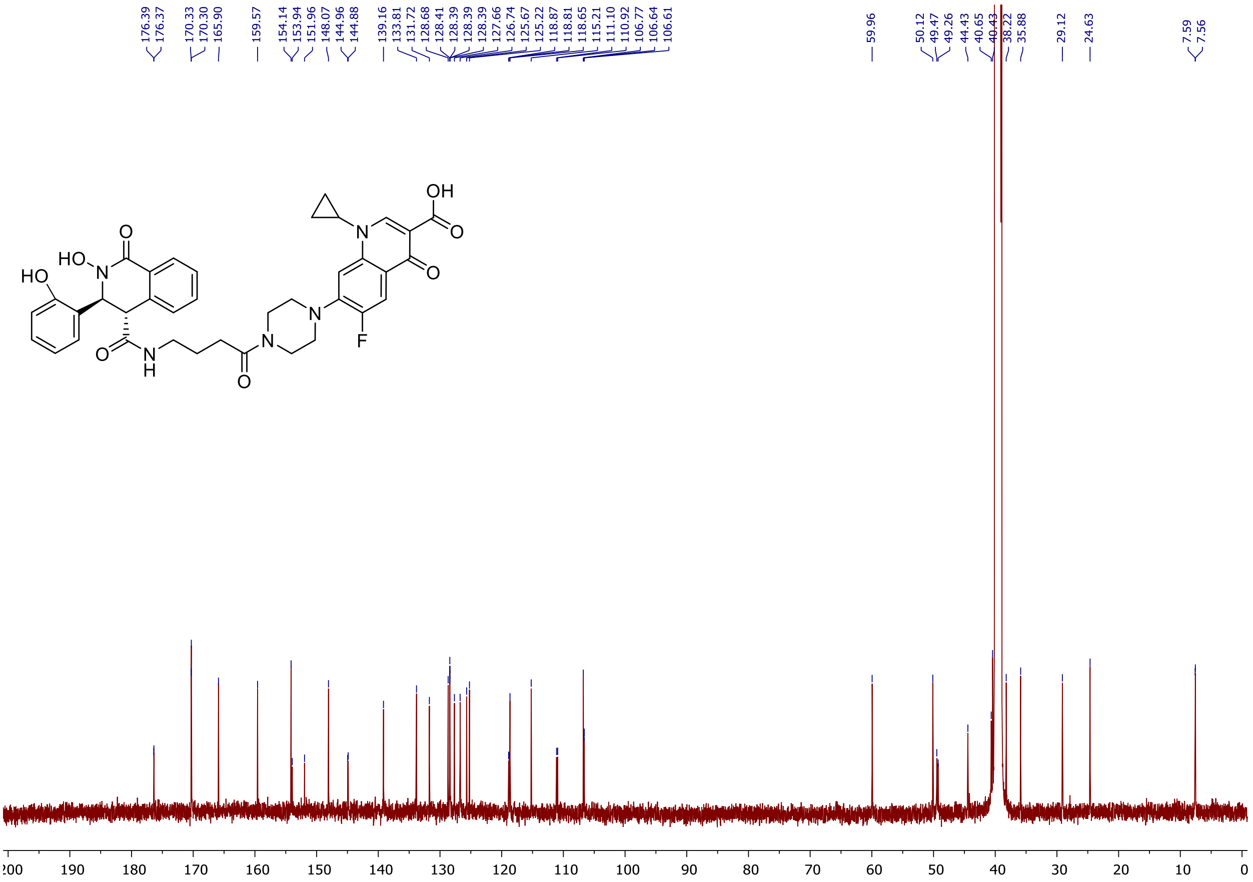

^{1}H and ^{13}C spectra of compound 8c

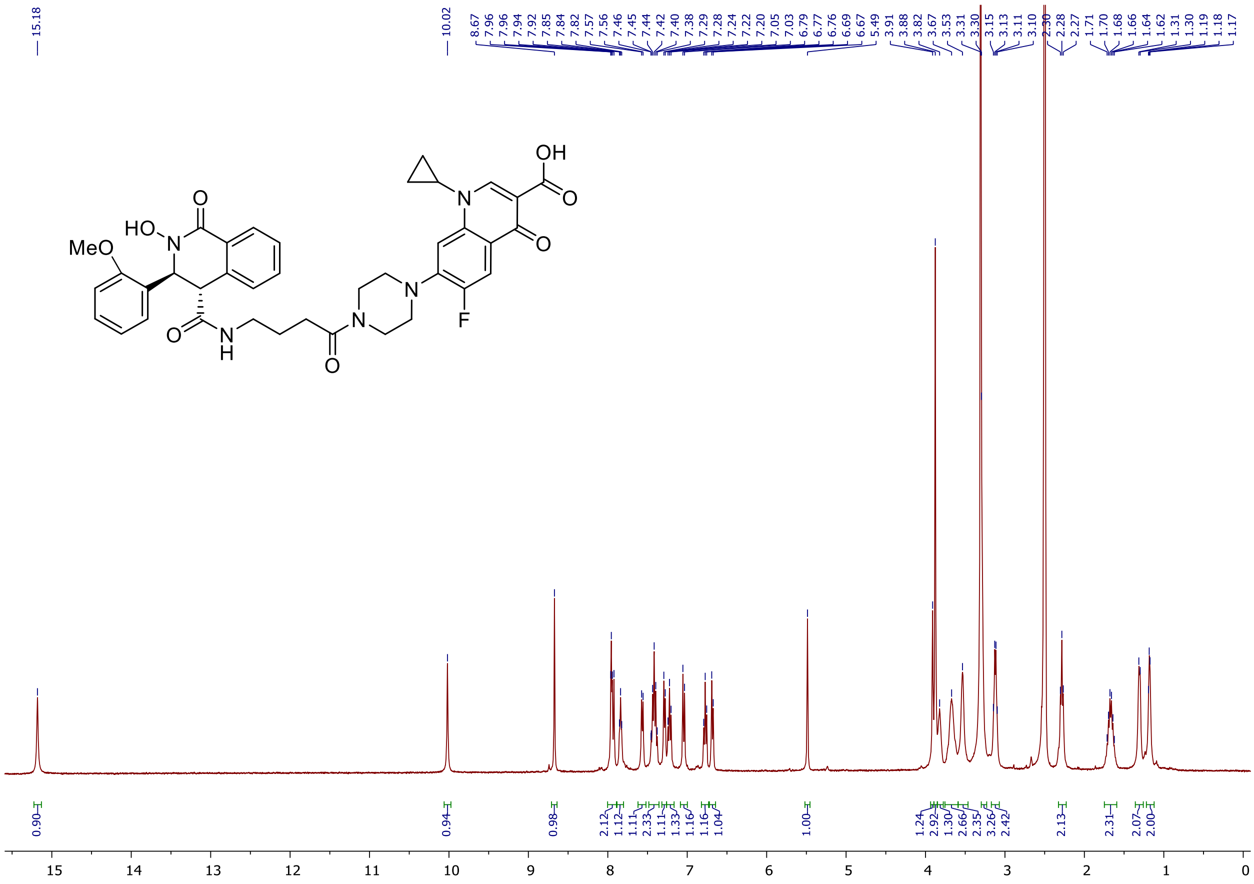

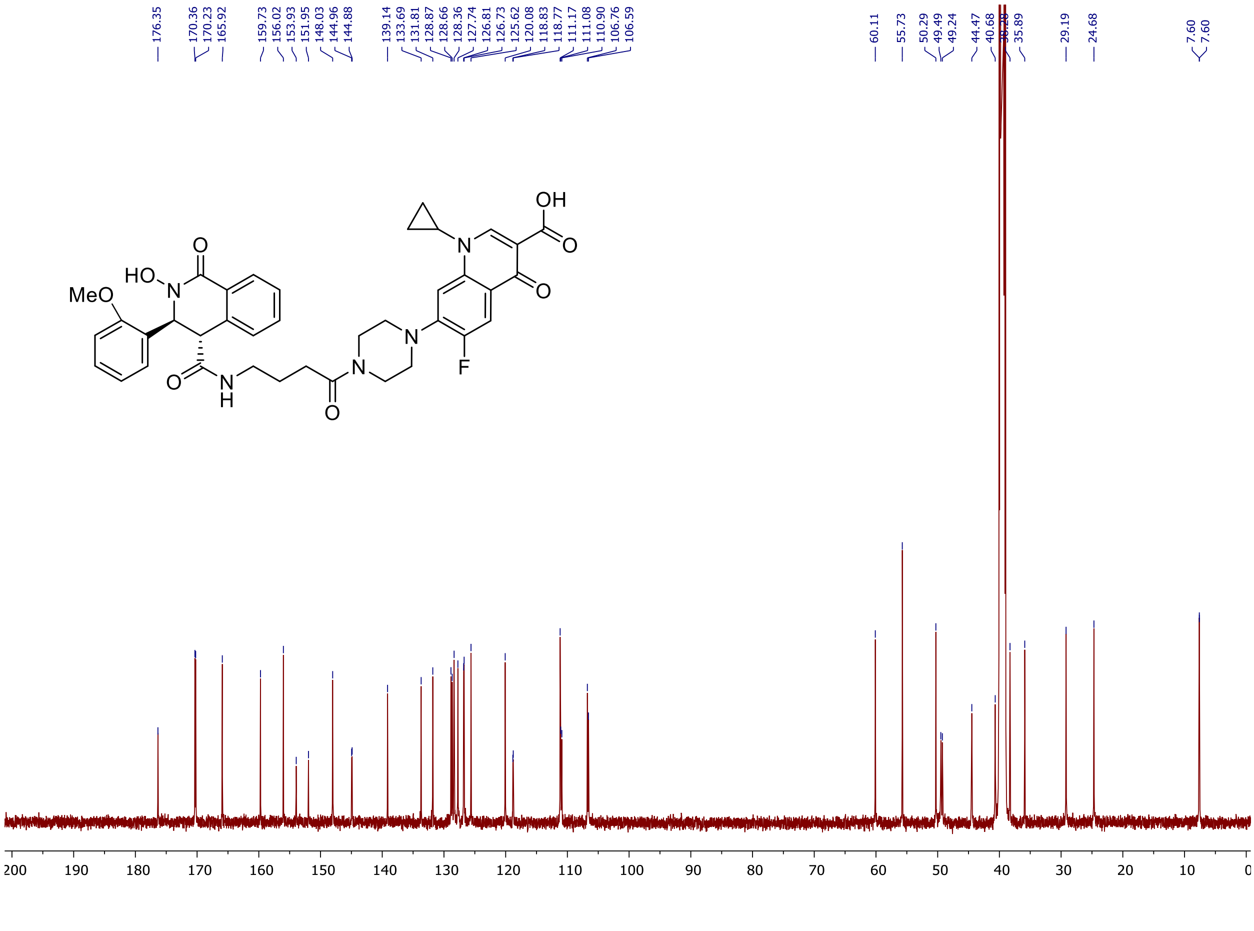

^{1}H and ^{13}C spectra of compound 8d

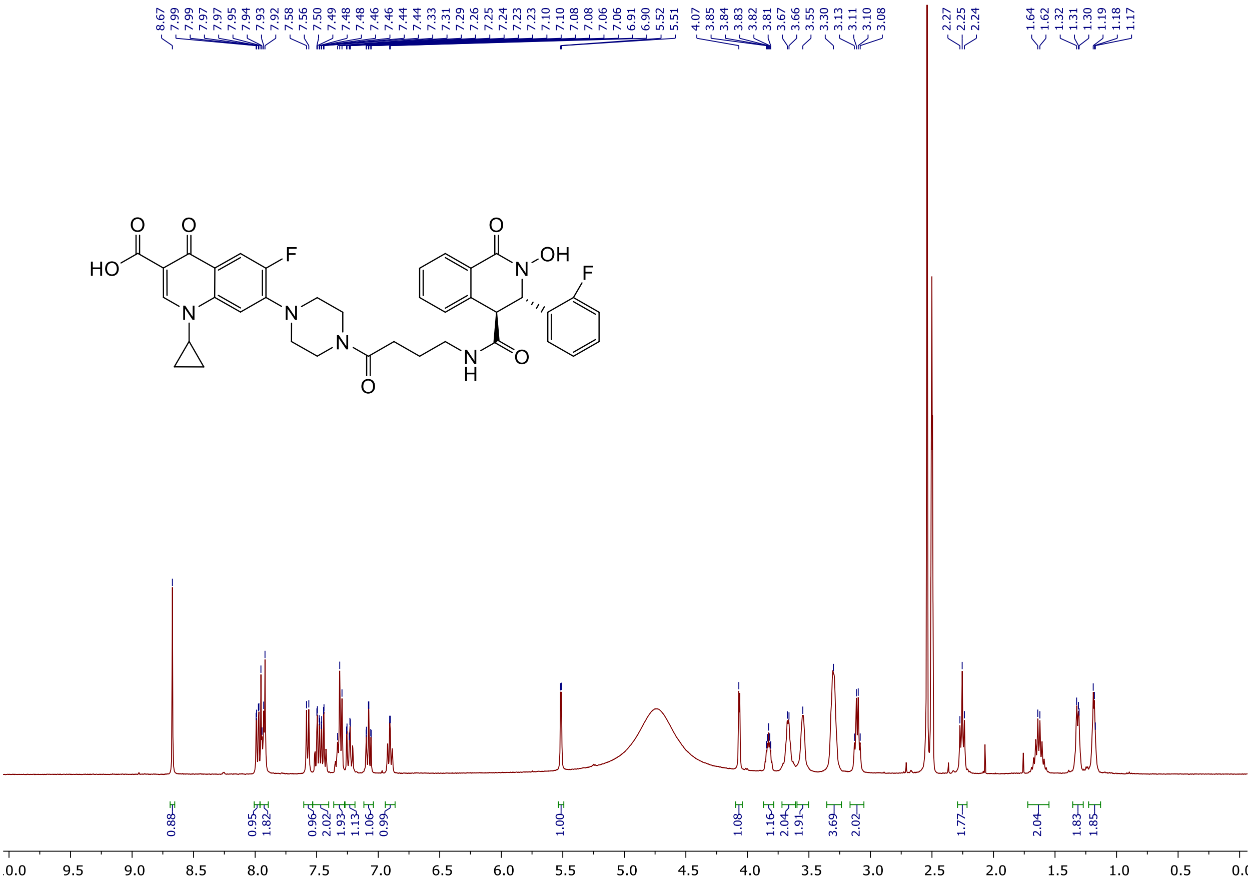

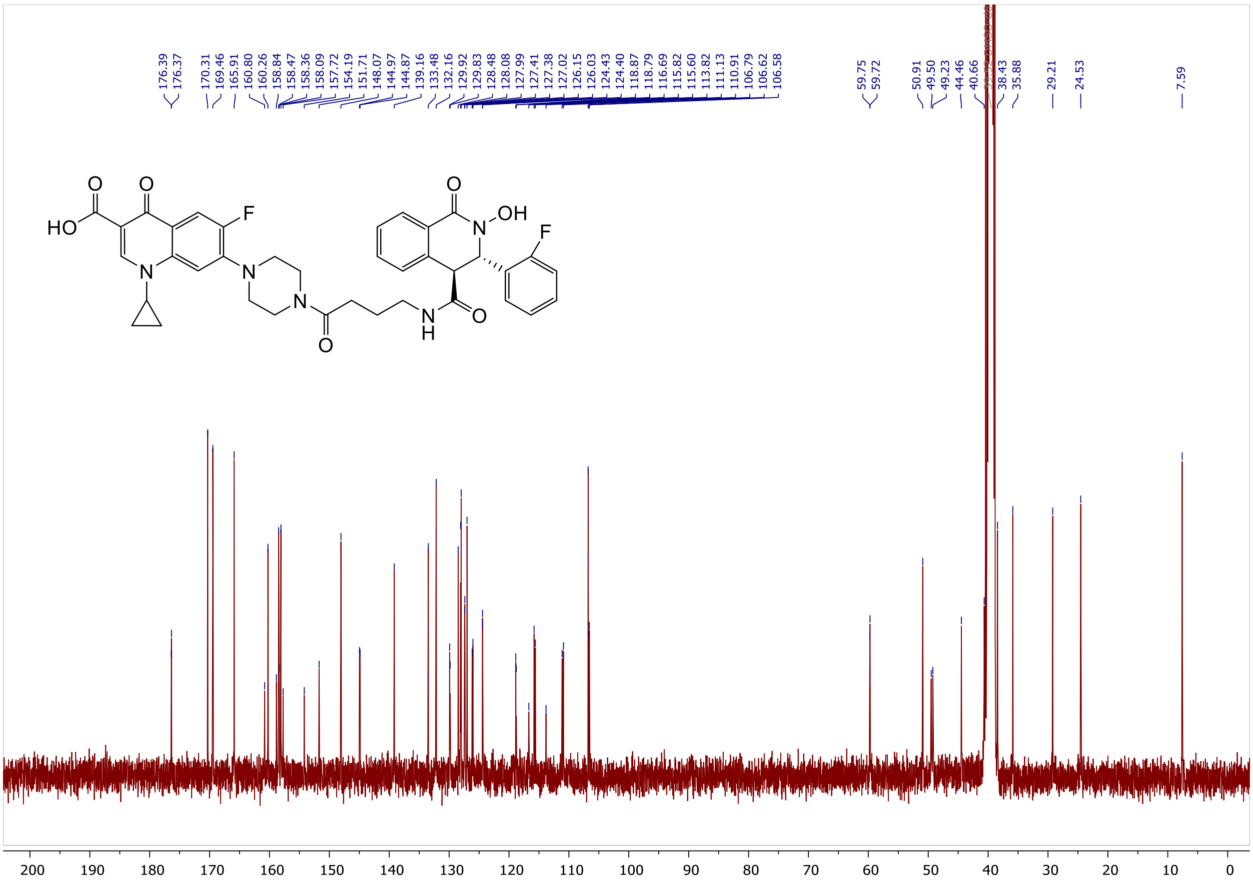

Structures of compounds **1a-z**

Compound	Structure	Compound	Structure
**1a**	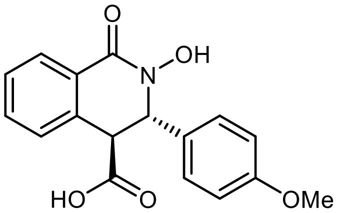	**1n**	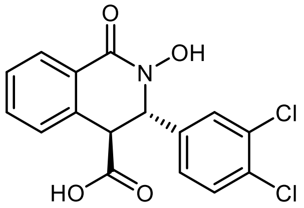
**1b**	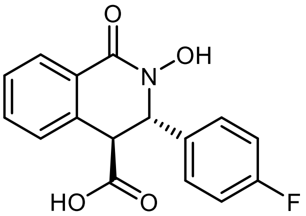	**1o**	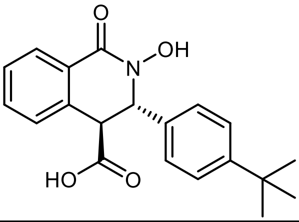
**1c**	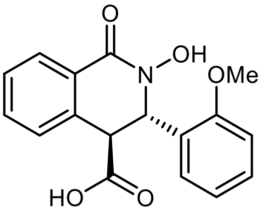	**1p**	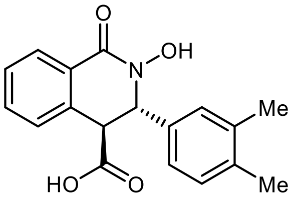
**1d**	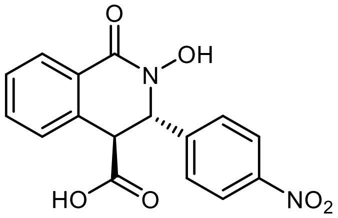	**1q**	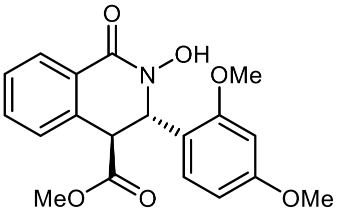
**1e**	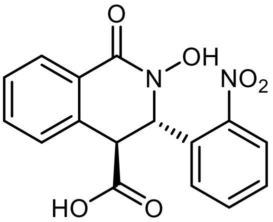	**1r**	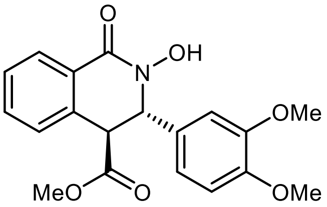
**1f**	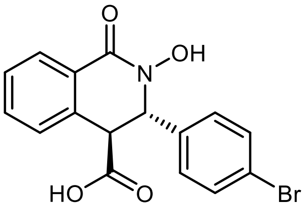	**1s**	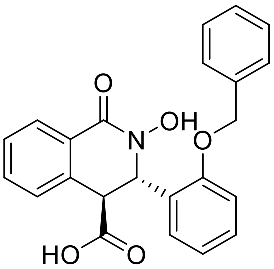
**1g**	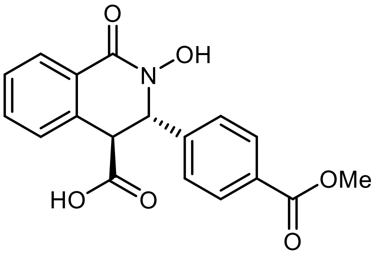	**1t**	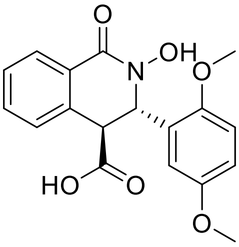

**1h**	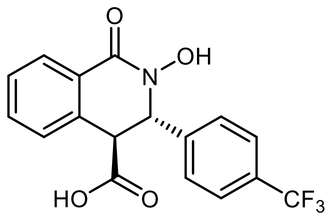	**1u**	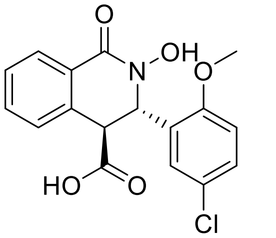
**1i**	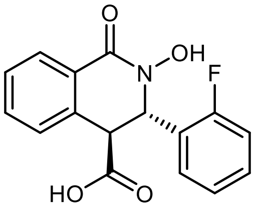	**1v**	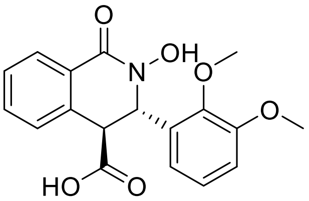
**1j**	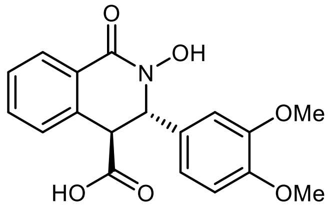	**1w**	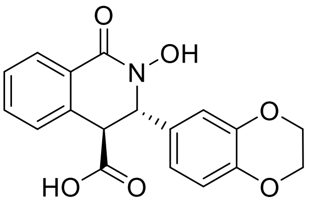
**1k**	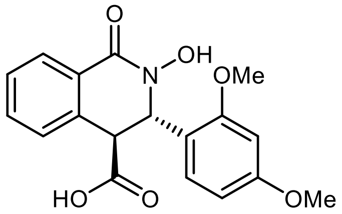	**1x**	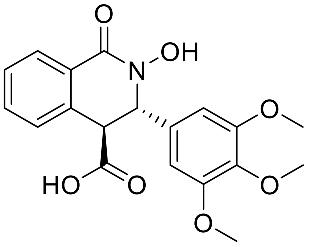
**1l**	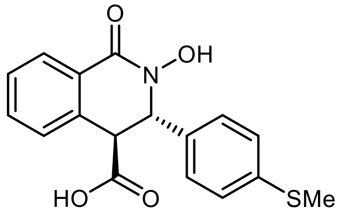	**1y**	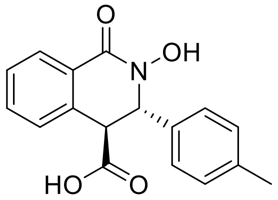
**1m**	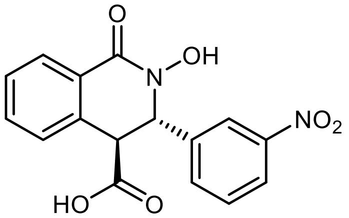	**1z**	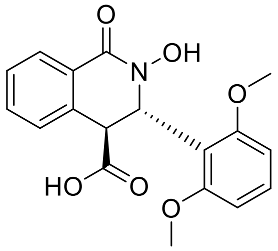

Figure S1. Growth of Δ*pvdF*Δ*pchA P. aeruginosa* mutant in the absence or presence of 10 μM and 100 μM of the different compounds in iron-restricted medium. The growth medium used was CAA and growth was followed by monitoring optical density (OD) at 600 nm at 30 °C.

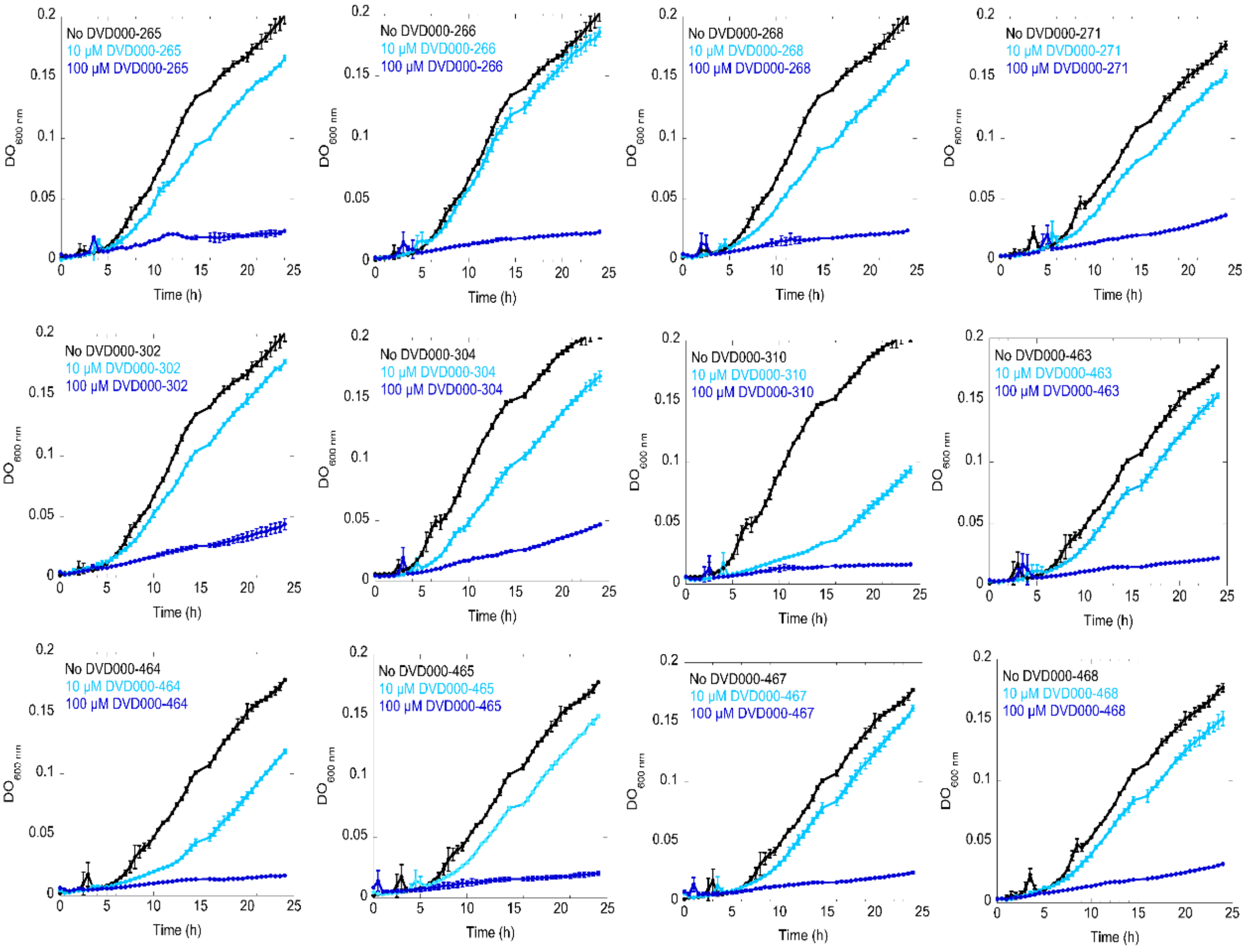

Legend: DVD000-265 (**1a**), DVD000-286 = DVD000-627 (**1c**), DVD000-268 (**1d**), DVD000-271 (**1z**), DVD000-302 (**1e**), DVD000-304 (**1i**), DVD000-310 (**1k**), DVD00-463 (**1t**), DVD000-464 (**1u**), DVD000-465 (**1v**), DVD000-467 (**1x**), DVD000-468 (**4**).
